# Deep Learning Framework for Atomic-Level Design and Presynthesis Prediction of Coinage-Metal Nanoclusters

**DOI:** 10.1021/acscentsci.5c01610

**Published:** 2026-01-08

**Authors:** Jiayi Wang, Chunwei Dong, Xiaochuan Gou, Shaopeng Fu, Peng Yuan, Xin Song, Mohammad Bodiuzzaman, Mutalifu Abulikemu, Wanyu Lin, Ren-wu Huang, Omar F. Mohammed, Di Wang, Osman M. Bakr

**Affiliations:** † Center of Excellence for Renewable Energy and Storage Technologies, Division of Physical Science and Engineering (PSE), 127355 King Abdullah University of Science and Technology (KAUST), Thuwal 23955-6900, Kingdom of Saudi Arabia; ‡ Innovation Institute of Carbon Neutrality, Department of Chemistry, College of Sciences, 34747 Shanghai University, Shanghai 200444, People’s Republic of China; ¶ Center of Excellence for Generative AI, Division of Computer, Electrical and Mathematical Sciences and Engineering (CEMSE), 127355 King Abdullah University of Science and Technology (KAUST), Thuwal 23955-6900, Kingdom of Saudi Arabia; § Department of Data Science and Artificial Intelligence, Department of Computing, 26680 The Hong Kong Polytechnic University, Hong Kong, China; ∥ Henan Key Laboratory of Crystalline Molecular Functional Materials, College of Chemistry, Pingyuan Laboratory, 12636 Zhengzhou University, Zhengzhou 450001, P.R. China

## Abstract

The atomically precise nature of coinage-metal nanoclusters (CMNs) enables systematic exploration of structure–property relationships and motivates application oriented inverse design. However, the synthesis of CMNs typically relies on trial-and-error methods, with atomic-level structures only revealed through crystallography (postsynthesis), posing a major challenge to the deterministic synthesis of predesigned cluster structures, which is known as **inverse synthesis**. Here, we introduce CoLiM, a deep neural network framework that predicts the chemical compatibility between the unexplored inorganic core and ligands *before synthesis*. CoLiM employs a dual-encoder architecture and is trained on a newly constructed dataset comprising 1,989 reported CMN structures, supplemented by an additional gas-phase cluster dataset. The optimal CoLiM model achieves an area under the curve (AUC) exceeding 0.83 on a held-out test set, outperforming all of the baseline methods. To demonstrate its practical utility, CoLiM is applied to address the long-standing challenge of achieving atomically precise structural tailoring. Starting from [Cu_20_Cl­(PET)_12_(PPh_3_)_4_­(MeCOO)_6_]^+^, we successfully performed single-atom editing on its inorganic core to synthesize [Cu_19_Cl­(PET)_12_(PPh_3_)_3_­(HCOO)_6_] guided by the prediction of CoLiM, validating the model’s generalizability under real experimental conditions. Our framework facilitates the inverse synthesis and precise atomic-level modification of nanoclusters, underscoring its substantial potential to accelerate rational nanocluster discovery.

## Graphical abstract

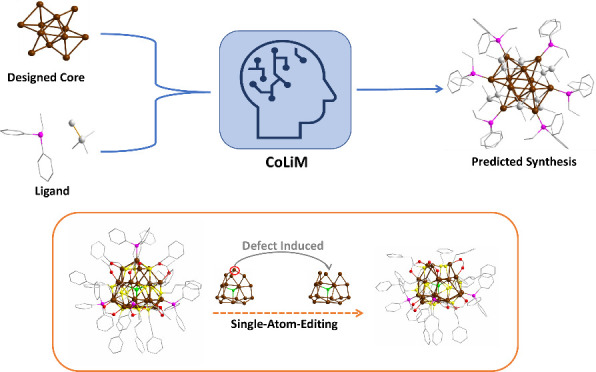

## Introduction

Coinage-metal nanoclusters (CMNs)—comprising copper, silver, gold, and their alloys—are a distinct class of nanomaterials characterized by their atomically precise structures and ultrasmall size (1–3 nm).^1–5^ These ultrasmall core–shell structures endow CMNs with distinctive molecule-like properties,^2^ making them invaluable across a range of applications, including catalysis, sensing, and imaging.^6–20^ The precise atomic arrangement, coupled with the versatility of their core and ligand-shell configurations, allows for tailored property customization.^6–8,21,22^ The composition of the inorganic core is known to influence the physical properties of the cluster,^2,23–26^ while the ligand shell plays a crucial role in determining its functionality.^2,4,13,27–29^ Due to the robust structure–property correlations, property-driven inverse design holds considerable potential for advancing application-specific CMNs. The inverse design of CMNs therefore involves two stages: first, identifying a target cluster structure from the desired properties via first-principles calculations,^30^ and second, synthesizing this predesigned structure—a step we term **inverse synthesis**. However, precise synthesis of CMNs with targeted atomic structures and compatible ligands still relies on trial-and-error approaches^26,28^ with structures confirmed only postsynthesis via single-crystal X-ray diffraction and mass spectrometry. This conventional experiment-characterization procedure restricts the speed and scope of exploration and significantly increases the resource costs of developing application-specific CMNs.

Recent advancements in data-driven methods, such as deep neural networks (DNNs), have demonstrated significant progress in assisting inverse design problems in molecular science and materials sciences.^31–41^ These technological strides offer an efficient and streamlined paradigm for exploring vast chemical spaces and assisting in sophisticated materials design. In particular, the integration of DNN in advancing the study of nanomaterials has also yielded promising outcomes. For instance, several studies employed DNNs to predict the properties of gas-phase clusters based on the structure descriptor (SD).^42–44^ To precisely determine the crystal structure, convolutional neural networks (CNNs) have been applied to determine the hydride locations.^45^ Moreover, graph neural networks (GNNs) have been employed to predict the formation energy of gold nanoclusters.^46^ Compared to conventional DFT methods, these deep learning (DL) based models make instantaneous inferences to predict clusters’ chemical and physical properties. Nevertheless, applying DNNs to aid the inverse synthesis of CMNs remains an unexplored frontier. The challenges are 2-fold: first, the inapplicability of DFT-generated datasets necessitates the manual collection of experimental data, and second, existing DL models inadequately represent the unique core–shell architecture of CMNs.

To tackle the raised challenges, we introduce a novel **Co**re-**Li**gands **M**atching model (CoLiM) along with a core-ligands matching dataset. CoLiM facilitates atomically precise inverse synthesis of new CMNs through predicting chemical compatibility of the inorganic core and ligands before synthesis experiments. The overview of the CoLiM framework for assisting inverse design is illustrated in Figure 1. CoLiM employs dual encoders to independently represent core configurations and ligand sets, predicting the compatible probabilities from their high-dimensional representations. CoLiM’s training pipeline consists of two stages. The first stage develops dual encoders projecting inorganic cores and ligands into a latent space encoding structural and chemical features; subsequently, CoLiM is fine-tuned using these pretrained encoders to predict core-ligand synthetic compatibility. To effectively train CoLiM, we constructed the first core-ligands matching dataset from 1,989 previously reported CMNs’ structures, sourced from the Cambridge Crystallographic Data Centre (CCDC),^47^ with meticulous data cleaning steps. The performance of CoLiM is assessed on the test set and compared with two categories of baseline methods: traditional machine learning (ML) models employing structural descriptors and GNN-based DL models. CoLiM with the pretrained encoder achieves an area under the curve (AUC) over 0.83, significantly surpassing all baseline methods.

**Figure 1:**
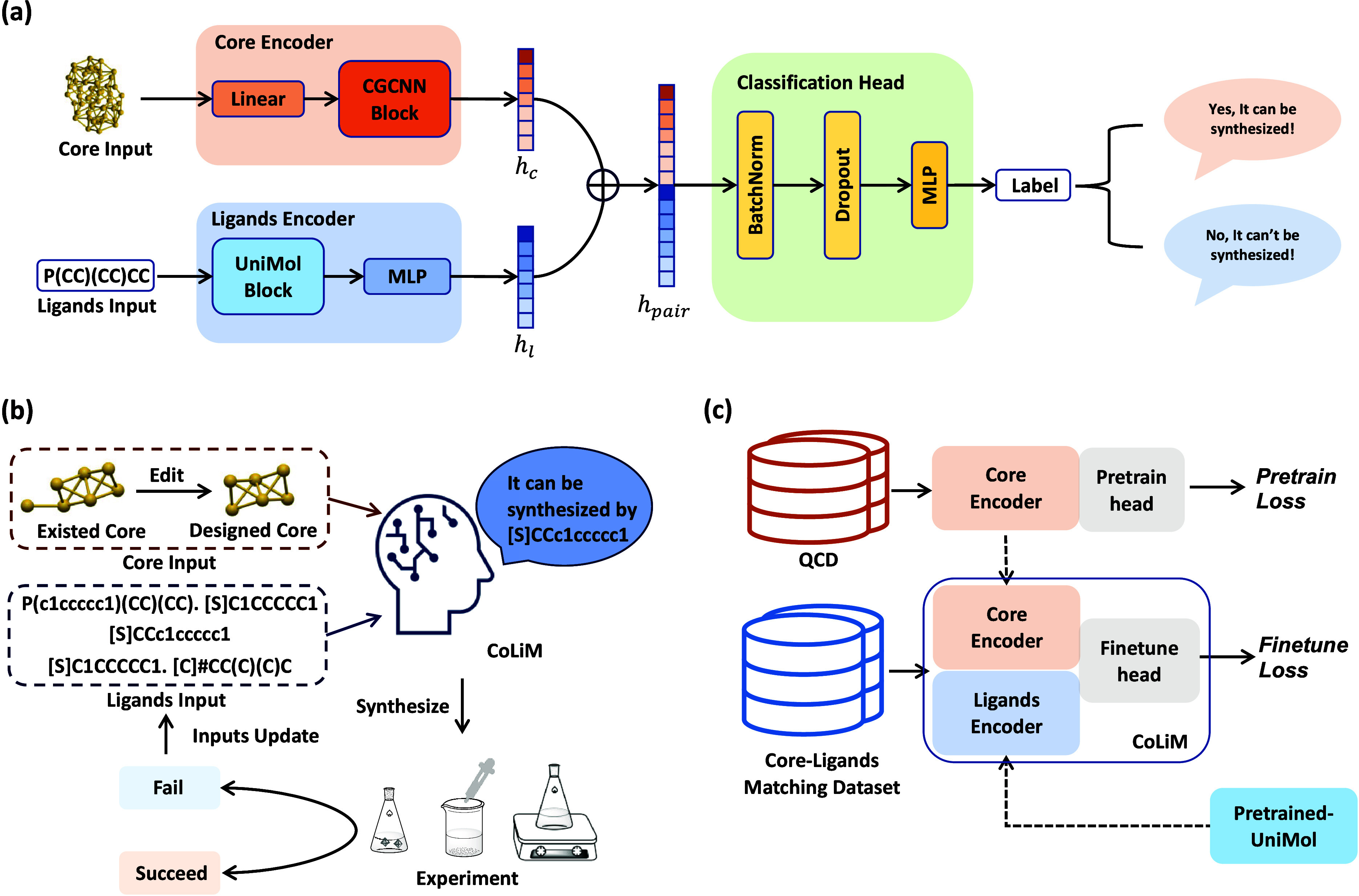
Overview of the CoLiM framework: (a) Model structure of CoLiM. The model employs a dual-encoder architecture where the inorganic core is processed using a core encoder to extract core feature *h*_c_, while the ligand input is encoded using a ligands encoder to obtain ligands feature *h*_l_. The concatenated pair feature vector *h*_pair_ is passed through a classification head to output the prediction. (b) A schematic diagram showing the application of CoLiM in facilitating the inverse synthesis procedure. A new core structure could be designed through modifications to an existing inorganic core, including the replacement, addition, and rearrangement of atoms. The redesigned core and provided ligand candidates are then fed into the CoLiM, to predict the most compatible ligands. A synthesis experiment is performed utilizing predicted ligands, while the final product is confirmed through crystallographic characterization. If it fails, an update on ligands and core input is required for another round of prediction until the final product is obtained. (c) The training pipeline of CoLiM. The training of CoLiM consists of two stages: encoder pretraining and CoLiM fine-tuning. The core encoder is pretrained using QCD, with the objective of predicting *E*_form_ or *E*_HOMO–LUMO_. The pretrained ligands encoder utilizes the pretrained UniMol. In the second stage, CoLiM is fine-tuned using the core-ligands matching dataset.

Despite the substantial challenges in achieving atomic-level tailoring of nanoclusters without disrupting their primary structure,^21,28^ such precise control remains essential for elucidating underlying structure–property relationships; thus, to demonstrate the capability of the proposed framework in addressing this longstanding issue, we conducted a representative case study. Based on newly synthesized [Cu_20_Cl­(PET)_12_(PPh_3_)_4_­(MeCOO)_6_]^+^, we performed single-atom editing and synthesized [Cu_19_Cl­(PET)_12_(PPh_3_)_3_­(HCOO)_6_] with one atom difference, utilizing ligands predicted by CoLiM. By integrating CoLiM with conventional experimental techniques, the proposed framework provides a promising strategy for the inverse synthesis of predesigned clusters and precise atomic-level structural tailoring.

## Results and Discussion

### Overview of the CoLiM Framework

Building on the rationale of inverse synthesis, CoLiM focuses on learning the intricate matching patterns from reported nanocluster structures, thereby making a presynthesis compatibility prediction of the unexplored inorganic core and ligand pairs. We define “chemically compatible” for a given core-ligand pair if the input core configuration could be synthesized utilizing the given ligand combinations using any synthetic methods under the existing experimental protocols.

Figure 1 illustrates the overview of the CoLiM framework, depicting the model architecture design, CoLiM-assisted inverse synthesis framework, and training pipeline. To capture the chemical and geometric information inherent in the core and ligand inputs, CoLiM utilizes a dual encoder architecture, as shown in Figure 1a. Specifically, upon receiving a pair of core configurations and ligands set in Simplified Molecular Input Line Entry System (SMILES) as input, CoLiM generates a core representation *h*_c_ and a ligands representation *h*_l_ through the core encoder and ligands encoder, respectively. Notably, we have shown that representation generated from UniMol is indeed permutation-invariant (Table S22). Therefore, in our settings, the representation is both insensitive to the order and ratio of different ligand molecules.

A pair representation *h*_pair_ is obtained by concatenating these two high-dimensional representations. The final predicted probabilities for the binary labels are obtained via a task-specific classification head composed of batch normalization, dropout, and fully connected layers. The architecture of CoLiM is motivated by the distinct elemental composition and geometric arrangement of the inorganic metal core and the surrounding protective ligands shell, ensuring alignment with the underlying learning objectives. By partitioning the inputs into separate representation spaces, CoLiM achieves improved modeling efficiency, enhancing its scalability and applicability to complex cluster structures and large-scale ligand screening tasks.

We propose a workflow that integrates CoLiM with laboratory synthesis methods to address the practical challenge of cluster inverse synthesis (Figure 1b). Initially, the inorganic core can be designed by modifying a reported core structure through atom substitution, removal, or addition. Alternatively, a new core configuration can also be designed from scratch. To synthesize the target cluster with the newly designed core, CoLiM is employed to predict compatible ligands by screening the candidate protective ligands provided by the cluster designer. Subsequently, synthesis experiments are conducted using the ligand combinations suggested by CoLiM to realize the designed structure. The inspection of synthesis results and crystallographic characterization determines whether the attempt is successful or the core structure and ligands inputs need to be revised for the next round of prediction.

### Dataset Construction

Recognizing the crucial impact of the quality of training data on the effectiveness of DL models,^48,49^ we meticulously constructed our dataset from published nanocluster structure data. The dataset construction involves a detailed four-step process (Figure S2). Initially, the Crystallographic Information File (CIF) for 2,500 compounds that are potential candidates for CMNs and contain the base elements gold, silver, and copper in certain stoichiometric ratios are sourced from the Cambridge Crystallographic Data Centre (CCDC). These raw data are processed through a rigorous data cleaning process to eliminate unqualified data, as detailed in the Method section. This meticulous filtration results in 1,989 high-quality cluster structure data, providing a solid foundation for training robust and reliable models. The collected cluster structures exhibit a broad size distribution (Table S10), indicating their structural diversity and potential versatility in various applications. More statistical analysis of the data distribution is further provided in the SI, including nuclearity distributions (Tables S13 and S14), the nanocluster size distribution (Figure S3), and the distribution of the ligand family (Table S15). For every structure, the inorganic core and its corresponding protective ligands are meticulously cataloged and stored in 3D coordinate format (XYZ file) and SMILES notation, respectively, facilitating the creation of the metal core library and protective ligands library. In case of multiligands protection, the SMILES of ligands set is constructed through concatenating with “.”. The statistical properties of the constructed core/ligands library are shown in Table S11 and Table S12, illustrating the proportion of multimetal alloying and multiligands protection in existing CMNs’ structures.

Given the constructed inorganic core and ligands library, the core-ligands matching dataset is then built through the data labeling process. We define the Positive data as those core-ligand pairs that are chemically compatible. To ensure the rigor of our definition, we define the Negative data for those pairs that are unable or less likely to form the core through this ligand set under the present experimental methods and protocol. Briefly, a pair of cores and ligands is labeled as Positive if the structure has been successfully synthesized and reported. Negative core–ligand pairs are generated by sampling from the filtered pool of negative ligands for each core structure, with details shown in the Methods section. The dataset comprises 3,978 samples, labeled as either positive or negative, with a balanced distribution of 1,989 samples in each class. Each sample contains one inorganic core structure and one set of ligands, with a maximum of 4 types of ligands. The final dataset is split into 80% training, 10% validation, and 10% testing for further model training and evaluation.

### Encoder Pretraining and CoLiM Fine-Tuning

To effectively train CoLiM, we constructed the training pipeline into two primary phases: encoder pretraining and CoLiM fine-tuning, as depicted in Figure 1c. At the pretraining stage, the core encoder is trained in a supervised manner using 63,015 gas-phase nanocluster data with labels obtained from the Quantum Cluster Database (QCD).^50^ QCD is the largest dataset of gas-phase nanoclusters containing DFT-calculated structural and physical properties encompassing 63,015 samples across 55 elements. The pretrained core encoder is obtained through optimizing the core encoder model on two specific regression tasks from QCD: prediction of the formation energy (*E*_form_) and the HOMO–LUMO gap (*E*_HOMO–LUMO_). The model is based on a GNN architecture, incorporating multiple GNN layers to generate high-dimensional representations of the input and a multilayer perceptron (MLP) for final energy prediction. We benchmarked the most widely adopted GNN models on the two pretraining regression tasks (Table S1). Among all evaluated models, the modified CGCNN^33^ exhibited the lowest mean absolute error (MAE) among all GNN models and was thus selected as the core encoder module for downstream CoLiM fine-tuning. Notably, the model trained specifically on *E*_*f*orm_ prediction is chosen for fine-tuning, with a detailed rationale for this selection provided in the subsequent sections.

Given that the protective ligands belong to the class of organic molecules, which have been extensively studied in the molecular representation learning task, we adopted the pretrained UniMol architecture^51^ as the ligand encoder module in CoLiM. UniMol, a widely used transformer-based model, has demonstrated excellent performance as a molecular encoder across various tasks.^51,52^ Compared to other deep learning-based molecular representation methods, UniMol is selected due to its superior capability to capture molecular features and its compatibility with our fine-tuning pipeline, as it takes SMILES representations as input. The pretrained core encoder and the Unimol block are subsequently integrated into the CoLiM model to fine-tune on the core-ligands matching dataset. During fine-tuning, the prediction task is treated as a binary classification problem, with CoLiM predicting labels based on the 3D coordinates of the inorganic core configurations and the SMILES representations of ligands. This novel training pipeline enables CoLiM to leverage knowledge from a broader range of related datasets, effectively mitigating performance limitations arising from the relatively small size of our custom-constructed dataset.

### Performance Evaluation

To comprehensively evaluate the overall performance of CoLiM, we benchmarked it against graph neural network (GNN) based DL methods and structure descriptor (SD) based ML methods. GNN-based models demonstrate remarkable capability in learning representations of molecules and atomic configurations.^33,53,54^ These models typically employ a message-passing neural strategy, treating atomic structures as graphs and leveraging node and edge features to encode spatial, chemical, and interaction-related information on the atomic systems.^53^ Considering the dataset size, CGCNN,^33^ SchNet,^54^ and GIN^55^ are selected as baseline models. To further demonstrate the advantages of our proposed method, we compared the CoLiM models with the SD-based ML methods. Specifically, Many-Body Tensor Representation (MBTR),^56^ Smooth Overlap of Atomic Positions (SOAP),^57^ and Atom-centered Symmetry Functions (ACSF)^58^ are chosen as representative SD-based descriptors. These descriptors transform atomic configurations and molecules into high-dimensional feature spaces, thus effectively encoding geometric, electronic, and chemical information.^44^ Subsequently, these features are utilized as inputs for an XGBoost model to perform property prediction or binary classification tasks. Since the fine-tuning task is formulated as a binary classification problem, we adopted the Area Under the Curve (AUC) and accuracy as primary metrics to evaluate the model’s discriminative ability and overall classification performance. Despite the balanced nature of our dataset, we also included precision, recall, and F1-score to provide a comprehensive assessment, particularly regarding the trade-offs between false positives and false negatives.

Table 1 summarizes the performance comparison among different models on the test set across all evaluation metrics. Among the SD-based ML methods, ACSF achieves the highest AUC (0.732), accuracy (0.666), and F1-score (0.662). For GNN-based DL methods, GIN exhibits the best performance, obtaining an AUC of 0.806, an accuracy of 0.738, and an F1 score of 0.749. The CoLiM model without pretrained encoders achieves a comparable performance to that of GIN, obtaining an AUC of 0.801, an accuracy of 0.725, and an F1-score of 0.729. Notably, the CoLiM model with the pretrained encoder outperforms all baseline models, achieving the highest AUC (0.830), accuracy (0.769), and F1-score (0.772) with lower standard deviations indicating better model stability. We provide additional robustness analyses—probability calibration (reliability diagram, Figure S13), bootstrap confidence intervals (Table S20), and confusion matrices for the held-out test (Table S21)—which together indicate stable and reliable predictions for CoLiM with the pretrained encoder. These results underscore the effectiveness of CoLiM over the previous SD- and GNN-based methods. To qualitatively visualize the predictive capability of our classification model, Figure S4 visualizes four representative test data samples: Ag_12_Cu_7_, Ag_38_Cl_6_, Au_12_Pd, and Au_8_, along with their corresponding input ligands and ground truth label, reflecting the model’s accurate discrimination among different classes.

**Table 1:** Performance Comparison of Baseline Models; The Best Results Are Highlighted in Bold

		metrics				
methods	model	AUC*↑*	accuracy*↑*	precision*↑*	recall*↑*	F1-score*↑*
SD-based ML	MBTR	0.706 ± 0.018	0.6464 ± 0.022	0.65 ± 0.141	0.654 ± 0.035	0.652 ± 0.028
	SOAP	0.720 ± 0.016	0.648 ± 0.022	0.666 ± 0.024	0.622 ± 0.037	0.646 ± 0.023
	ACSF	0.732 ± 0.019	0.666 ± 0.013	0.672 ± 0.023	0.658 ± 0.026	0.662 ± 0.021
GNN-based DL	CGCNN	0.753 ± 0.033	0.725 ± 0.018	0.674 ± 0.012	0.828 ± 0.03	0.7414 ± 0.017
	SchNet	0.698 ± 0.026	0.636 ± 0.015	0.655 ± 0.059	0.747 ± 0.015	0.691 ± 0.05
	GIN	0.806 ± 0.015	0.738 ± 0.009	0.711 ± 0.014	**0.798** ± **0.001**	0.749 ± 0.008
CoLiM	CoLiM(Scratch)	0.801 ± 0.027	0.725 ± 0.018	0.712 ± 0.011	0.7526 ± 0.017	0.729 ± 0.020
	CoLiM(Pretrain)	**0.830** ± **0.016**	**0.769** ± **0.013**	**0.755** ± **0.456**	0.797 ± 0.531	**0.772** ± **0.470**

To further assess the model’s generalization ability, we evaluated CoLiM with pretrained encoders on a set of recently reported structures that are not included in our dataset. A complete inventory of external CMNs in the external test is provided in Table S16. Following the same preprocessing protocol used for the in-domain data, each external data sample is decomposed into core and ligand sets. Since every cluster in the external test has been experimentally synthesized, the correct behavior is to output a logit that maps to a probability greater than 0.5, corresponding to a “synthesizable” label for all samples. Figure 2 visually compares the predicted probabilities from CoLiM with those from the strongest SD-based baseline (ACSF) and the strongest GNN-based baseline (GIN). Each data sample, represented by its short name and structural visualization, is evaluated based on the predicted probabilities of assigning a Positive label. CoLiM demonstrates superior performance, successfully classifying 8 out of 10 external data. Notably, CoLiM (Pretrain) shows competitive predictions even in challenging scenarios where ACSF and GIN exhibit lower performance, such as those for Cu_26_Se_12_ and Cu_8_. These findings highlight the model’s ability to capture the intricate structural patterns of metal clusters and confirm that the pretraining strategy effectively extends CoLiM’s applicability beyond its original training domain.

**Figure 2:**
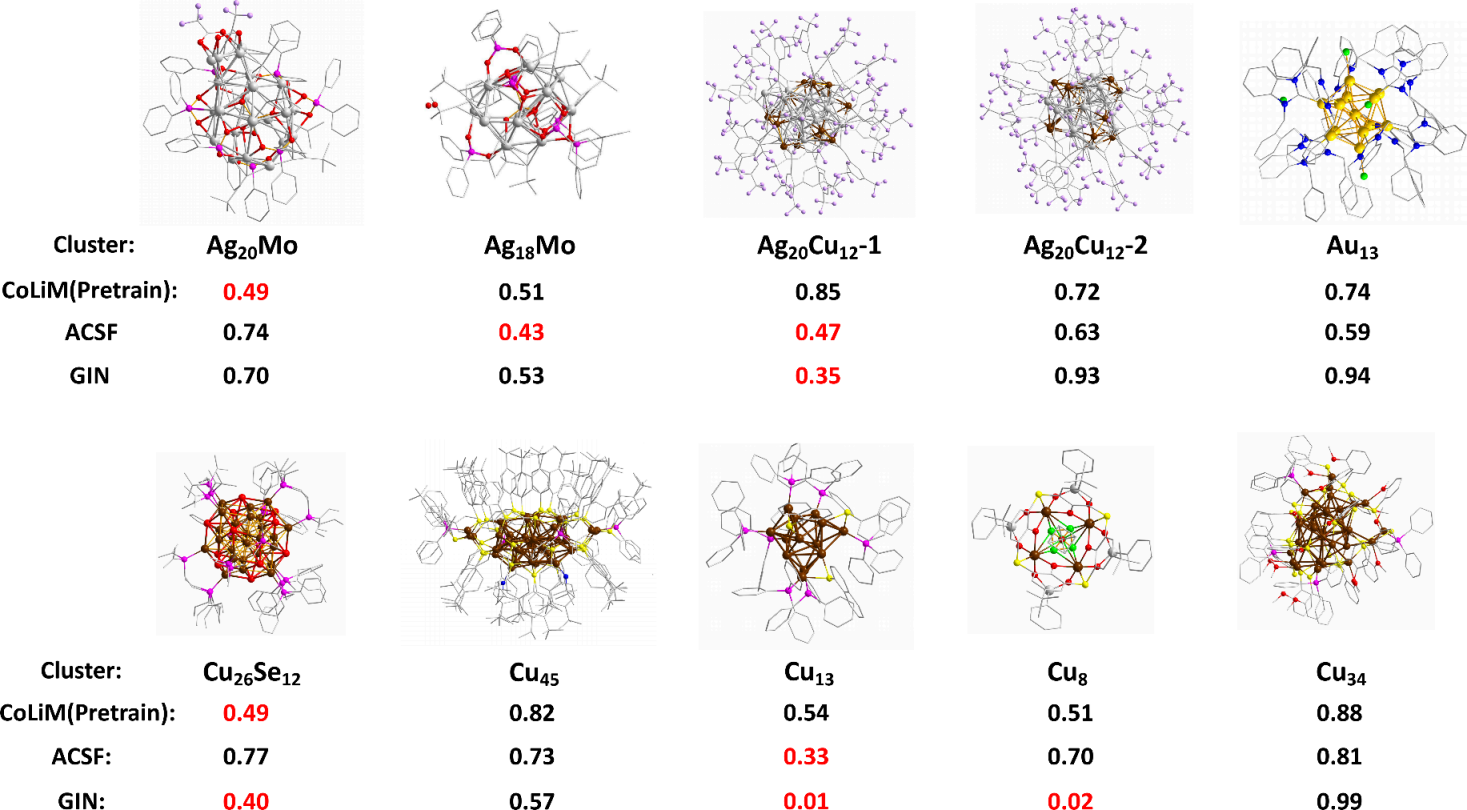
Prediction results for the external test data of CoLiM (Pretrain), ACSF, and GIN. The probabilities of assigning the Positive label are calculated by the models for the split inorganic core and ligands input, which is shown along with the cluster short name.

### Knowledge Gained from Encoder Pretraining

To quantify the performance gains offered by the pretrained encoder, we conducted an additional investigation into both versions of the CoLiM models. First, we analyzed the latent space learned by CoLiM with and without pretraining. Both models generated high-dimensional embeddings for all samples in the core–ligands matching dataset, after which we visualized the embeddings using t-SNE dimensionality reduction.^59^ Figure 3 displays the t-SNE embeddings, with class 0 shown in blue and class 1 shown in orange. Figure 3a (with pretraining) contains two compact, well-separated clusters, indicating that the encoder extracts strongly discriminative features for the two classes. Figure 3b (without pretraining) reveals a much more entangled distribution with substantial class overlap, reflecting weaker separability. This marked distinction underscores how pretraining sharpens the latent space and markedly improves class discrimination. This phenomenon is further supported by the Davies-Bouldin index (DBI)^60^ and Calinski-Harabasz index (CHI),^61^ where the pretraining model achieves a lower DBI of 23.49 and higher CHI of 6.15, indicating better cluster compactness and separation, compared to the model without pretraining, which has a DBI of 60.40 and a CHI of 0.97 (Figure S5).

**Figure 3:**
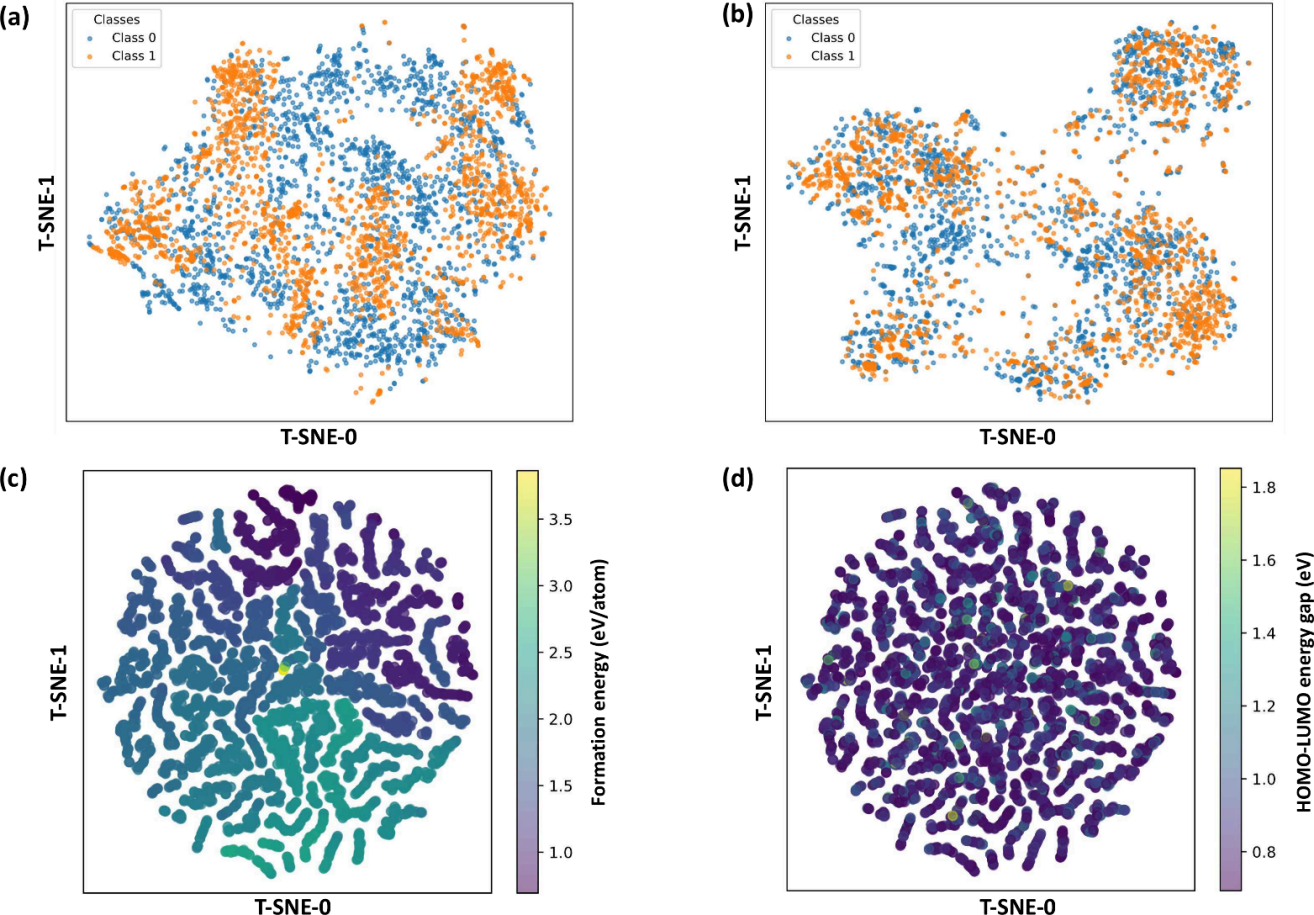
Knowledge gained from encoder pretraining. (a, b) T-SNE dimension-reduced visualization of pair representation from the core-ligands matching dataset generated by CoLiM with (a) and without (b) the pretrained encoder. (c, d) T-SNE dimension-reduced visualization of representation from pretrained core encoder pretrained on two regression tasks in QCD dataset: (c) formation energy (*E*_form_) prediction, and (d) HOMO–LUMO energy gap (*E*_HOMO–LUMO_) prediction.

To gain deeper insights into the knowledge acquired during core encoder pretraining, we visualized the hidden layer representations for its two pretraining tasks on the QCD dataset. Focusing first on the *E*_form_ prediction task, Figure 3c and d visualizes the formation energy (*E*_form_, eV/atom) across the QCD test set using color to represent the energy distribution. The close clustering of samples with similar *E*_form_ values reveals that the learned representations preserve energy-related properties, demonstrating the model’s effectiveness in capturing and structuring the underlying energy relationships. Second, we visualized the representations generated by models optimized for the *E*_HOMO–LUMO_ prediction task. The effectiveness of this model appears limited, as the data points exhibit dense clustering and poor separation. This indicates that the latent space representations lack clear discrimination between different HOMO–LUMO energy gap ranges. The resulting overlap and absence of distinct clusters suggest that the learned representations fail to effectively differentiate samples based on their HOMO–LUMO gap values, limiting their utility for downstream tasks. This visualization aligns with the higher MAE observed for *E*_HOMO–LUMO_ prediction on the QCD test set (Table S1). Furthermore, setting formation energy as the target is a direct thermodynamic proxy for synthetic stability, forcing the encoder to model interatomic interactions and elemental contributions that govern low-energy configurations. Consistent with this rationale, as this pretraining yields structured, energy-aware latent spaces and superior separability, whereas HOMO–LUMO gap pretraining produces entangled embeddings and higher prediction error—properties more reflective of optical/electronic behavior than stability. These observations support *E*_form_ as a more faithful inductive bias for our downstream objective.

### Case Study on Copper-Chloride Clusters

Precise atomic manipulation of nanomaterials is crucial for gaining profound insights into their structural and functional properties, enabling researchers to correlate atomic configurations with emergent properties. For example, the removal of a single atom from a cluster creates a point defect, which can significantly alter the electronic structure and consequently enhance or modify its catalytic activity.^21,62^ Such atomic-level structural modifications can dramatically alter the electronic environment around the active sites. This level of structural control accelerates the development of high-performance, application-specific nanomaterials. However, atomic-level tailoring of nanoclusters remains particularly challenging due to the intricate entanglement between the cluster core and its ligand shell. In this context, effectively pairing a desired core configuration with suitable ligands is essential, and CoLiM shows significant promise for accurately screening appropriate ligand combinations.

In this case study, we aimed to demonstrate the capability of the CoLiM-assisted inverse synthesis framework by atomically editing the inorganic core structure to introduce a point defect. We first synthesized a novel copper cluster, [Cu_20_Cl­(PET)_12_(PPh_3_)_4_­(MeCOO)_6_]^+^ (PET: 2-phenylethanethiol, PPh_3_: triphenylphosphine), abbreviated as Cu_20_. This new cluster is synthesized based on our previously reported nanocluster through ligand-exchange reactions,^63^ as experimental details in the Methods section. The light yellow crystals of Cu_20_ are obtained in the mixed solvents of chloroform and hexane within 1 day in the presence of acetic acid. Cu_20_ crystallizes in cubic *Pa*3̅ space group, and the structure is shown in Figure 5a, which is comprised of 20 Cu atoms, 12 PET, 6 acetates, 4 PPh_3_, and one centered chloride ion. The centered chloride ion is surrounded by four Cu atoms, as shown in Figure S6, suggesting the inverse coordination cluster character of Cu_20_. As far as we know, Cu_20_ is the largest inverse coordination cluster based on Cu and the halide. The other 16 Cu atoms form a large tetrahedral cage. The 6 acetates and 4 PPh_3_ are coordinated to the Cu atoms on the 6 edges.

We then introduced a point defect into the inorganic core of Cu_20_. Specifically, as illustrated in Figure 4, the new core structure, Cu_19_, is designed by removing one copper atom located at the apex of a tetrahedral vertex, thus introducing a defect site within the original cluster structure. Subsequently, we attempted the synthesis of this Cu_19_ cluster with the assistance of CoLiM. Based on the synthesis conditions for Cu_20_, several potential ligand candidates are selected, as shown in Table S18. Using these ligand sets and the atomic coordinates of Cu_19_, CoLiM predicted compatible probabilities, which are shown with corresponding ligand IDs in Figure 4b. To assess the robustness and stability of our predictions, inference was repeated using three independent models trained with identical hyperparameters but different random seeds. The resulting predictions are summarized in the heatmap shown in Figure S6. Guided by the prediction results, we experimentally synthesized the Cu_19_ cluster using two ligand sets: T1 (triphenylphosphine, 2-phenylethanethiol, and formic acid) and T5 (diphenyl-2-pyridyl phosphine, 2-phenylethanethiol, and formic acid), as illustrated in Figure 4c. The Cu_19_ cluster was successfully synthesized by using ligand combination T1, in which formic acid notably replaced acetic acid from the original Cu_20_ synthesis. In addition, we present comprehensive structural and spectroscopic characterization of two metal nanoclusters, Cu_20_ and Cu_19_. Figure 5b and f displays the ESI-MS spectra for Cu_20_ and Cu_19_, highlighting their mass-to-charge ratios with a sharp peak indicating high purity. Figure 5c and g compares the experimentally obtained isotopic distribution patterns with the corresponding theoretical simulations, exhibiting close agreement and thus validating our compositional assignments. Moreover, Figure 5d and h presents the UV–vis absorption spectra of Cu_20_ and Cu_19_ in chloroform solution, confirming their distinct optical properties, stability, and consistent electronic structures under experimental conditions. Collectively, these results confirm the successful atomic-level tailoring of the nanoclusters with the aid of the CoLiM framework and demonstrate its capability to introduce precisely engineered point defects.

**Figure 4:**
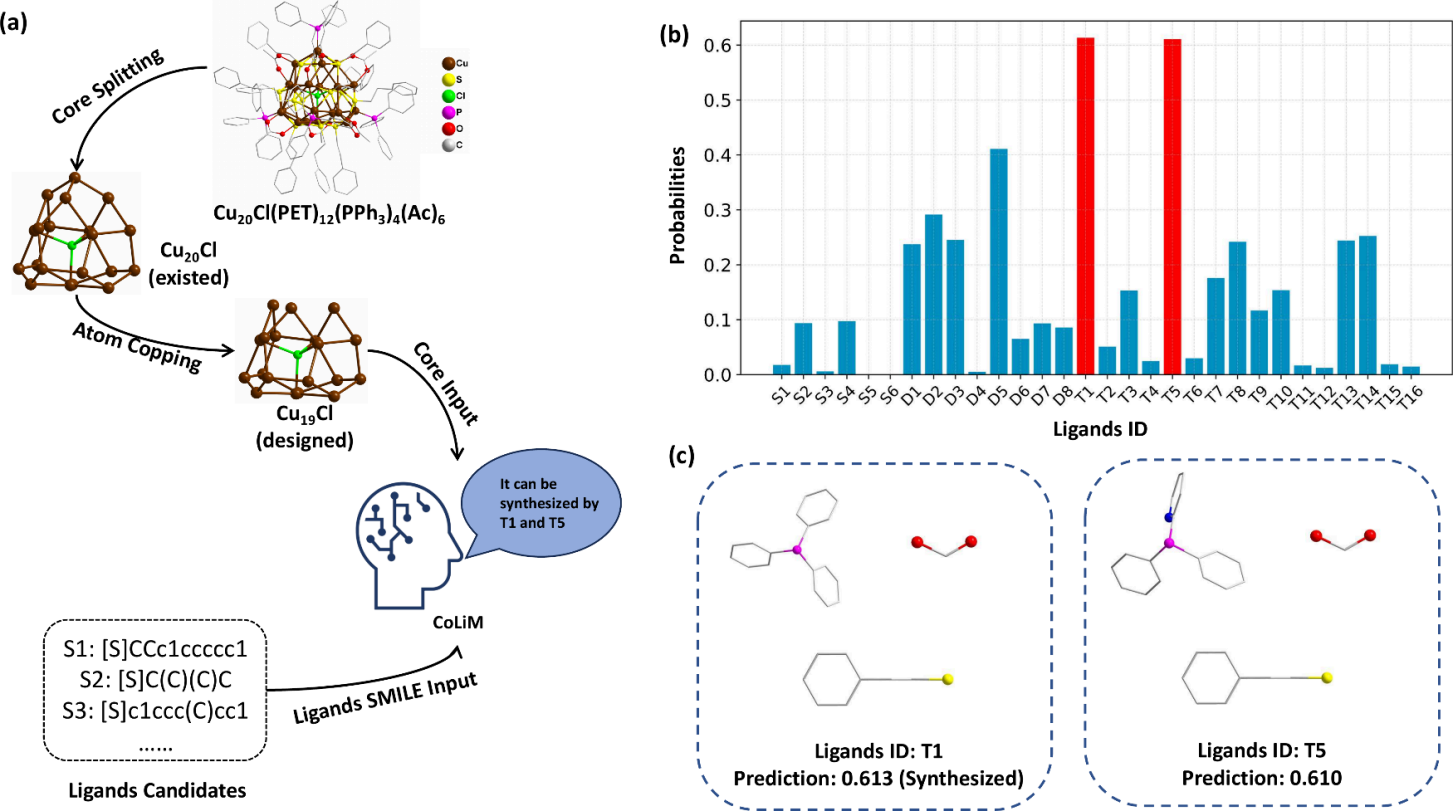
(a) Schematic illustration of the atomic-level structural tailoring process applied to the cluster [Cu_20_Cl­(PET)_12_­(PPh_3_)_4_(Ac)_6_]^+^. The core fragment, Cu_20_Cl, is extracted from the original cluster structure and subsequently edited to create the modified core configuration Cu_19_Cl. The designed Cu_19_Cl core, along with candidate ligand combinations, is provided as input to CoLiM, which then infers suitable ligands. (b) Predicted probabilities of ligand combinations. (c) Experimental validation through laboratory synthesis of nanocluster with Cu_19_Cl employing ligands sets T1 and T5, with successful synthesis with T1.

**Figure 5:**
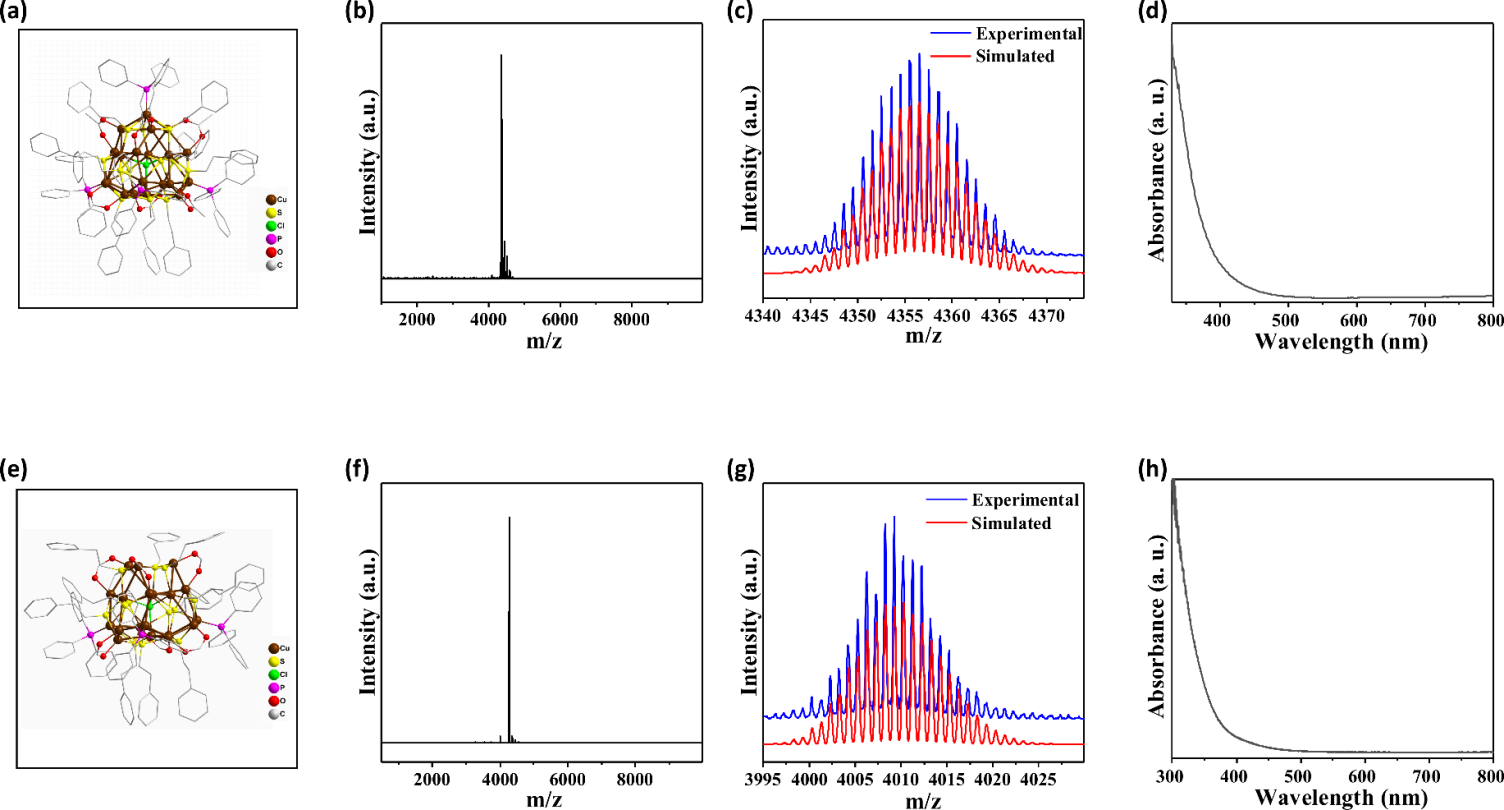
(a, e) Side view of Cu_20_ and Cu_19_. (b) ESI-MS spectra for Cu_20_. (c) Experimental (blue) and simulated (red) isotopic distribution patterns of [Cu_20_Cl­(PET)_12_(PPh_3_)_4_­MeCOO)_6_]^+^. (d) UV/vis absorption spectra of Cu20 in chloroform. (f) ESI-MS spectra for Cu_19_. (g) Experimental (blue) and simulated (red) isotopic distribution patterns of [Cu_19_Cl­(PET)_12_­(PPh_3_)_3_(HCOO)_6_]. (h) UV/vis absorption spectra of Cu_19_ in chloroform.

## Conclusions

In summary, we developed CoLiM, a predictive framework designed to address the inverse synthesis by accurately identifying compatible protective ligands for specific inorganic cores. CoLiM demonstrates excellent performance on core-ligand compatibility prediction tasks. A key finding of our work is that pretraining the encoder using relevant external datasets significantly mitigates the constraints typically posed by limited or narrowly varied experimental datasets. This novel pretraining strategy substantially improves CoLiM’s performance, allowing it to achieve an average AUC of 0.83, notably surpassing baseline models. To demonstrate its practical utility, we conducted a detailed case study focused on the precise structural manipulation of copper nanoclusters. Starting from a newly synthesized Cu_20_ cluster, we successfully performed atomic-level editing to introduce a single point defect and synthesized the Cu_19_ cluster using ligand combinations predicted by CoLiM. Looking ahead, coupling CoLiM with physics-based (DFT) or ML property predictors could enable a closed-loop workflow that begins from a target property specification and proceeds through structural modification such as core editing or ligand substitution, in-silico property screening, and a synthesizability check, ultimately yielding experiment-ready candidates while substantially reducing trial-and-error in the design of application-specific nanoclusters.

## Methods

### Preliminaries

The objective of our model is to determine the chemical compatibility between an inorganic core and a set of ligands, which is formulated as a binary classification problem. Given the atomic coordinates input of an inorganic core, we first represent it as a graph G={V,E} , where $V={\left\{v_{i}\right\}}_{i=1,...,n}$ is constitutive of a set of nodes (atoms) in a graph and $e_{i,j}=\left\langle v_{i},v_{j}\right\rangle \in E$ representing edge between nodes (bonds). A set of ligands represented in the SMILES representation S along with the graph G is fed to a model which predicts a binary label *y* ∈ {0, 1}, where *y* = 1 indicates compatible or “synthesizable” and *y* = 0 indicates not-compatible:

f:(G,S)→Y

where *f* is the proposed model. The objective is to establish a predictive framework that can identify synthesizable ligands for the given inorganic cores, enabling efficient screening and selection in inverse synthesis workflows. In the core encoder pretraining stage, the learning objective is defined as a value regression problem. Given the core input, the core encoder model is designed to map node and edge embeddings to a target property value *E*, such as *E*_form_ and *E*_HOMO–LUMO_:

$$f_{c}:\left(V,E\right)\to E$$

where *f*_c_ is the model for core encoder pretraining.

### Construction of Core-Ligands Matching Dataset

First, we downloaded the atom coordinate files (mol2) from CCDC by applying a filtered search for three coinage metals: gold (Au), silver (Ag), and copper (Cu), with stoichiometry ranging from 4 to 101. We subsequently undertook a series of data cleansing steps to enhance the dataset’s quality: 1. Structures that cannot be classified as CMNs are first excluded, such as salts and extended networks. 2. The target nanocluster molecules are then extracted by removing surrounding ligand parts which generated from CIF to mol2 file conversion. 3. Eliminating duplicate molecules in cases where multiple molecules are present within a single file. These data cleaning steps result in a total of 1,989 structures, which are further split into a separate inorganic core library and ligands library. We then constructed the core-ligands matching dataset based on these two libraries. Each data point in the dataset consists of two parts along with one assigned label: the 3D atomic coordinates of the core structure and a string representing the ligands in SMILES notation. For example, a nanocluster with the CCDC ID: 2223717, which comprises a core structure of Cu_58_ and two types of protective ligands (PPh_3_ and PET), is represented by Cu_58_ with the SMILES string P­(c1ccccc1)­(c1ccccc1)­c1ccccc1.[S]­CCc1ccccc1, and is labeled as positive. We first constructed a chemically informed pool of negative ligands for each metal core. Negative core–ligand pairs are then generated by randomly sampling from this pool in order to avoid additional manual cherry-picking of specific negative examples. For every metal core in the core library, cores were first classified according to whether an identical core geometry (i.e., same nuclearity and three-dimensional atomic arrangement, not merely the same nuclearity) has been reported with different ligand shells: (i) type I cores, for which no such alternative ligand sets are found, and (ii) type II cores, for which identical cores are stabilized by multiple ligand sets. For type I cores, current synthetic evidence suggests that they can be obtained only from specific ligand combinations. To avoid generating false negatives, ligands are removed from the candidate ligand library if they satisfy the following criteria:

1. The ligand shares the same functional group as the positive (experimentally observed) ligand.
2. The absolute difference in the number of carbon atoms between the ligand and the positive ligand is ≤7 .

For type II cores (e.g., structurally robust metal frameworks that can be synthesized with diverse ligand environments such as Au_25_ and Au_13_), stricter filtering rules are applied. In this case, a ligand is excluded from the negative pool if:

1. It has the same functional group as all positive ligands with this type of core.
2. The difference in the number of carbon atoms between the ligands and the positive ligands is ≤7 .

As for type II cores, the left ligands are randomly combined to form potential candidates with a different number of ligand types compared with the positive sample. Exception is made for ligands that satisfy these criteria but are explicitly reported in the literature or experimental logs as unable to produce the corresponding metal core, such as introducing new ligands resulting in a different core (e.g., through ligand exchange in our case study); such ligands are retained and randomly paired with other ligands to form negative samples. Once the 3978 data are constructed, we split it into 80% training, 10% validation, and 10% testing. We applied a grouped split at the core–ligand level: all entries that share the same metal core geometry (nuclearity) and the same ligand set are treated as a single group and assigned in their entirety to either the training and validation sets or the test set. Consequently, no identical or closely similar core–ligand combinations appear in both training/validation and test sets.

### Metrics

The evaluation of model performance on pretraining and fine-tuning tasks is conducted using a set of metrics: area under the curve (AUC), accuracy, recall, precision, F1 score, and mean squared error (MSE).

- **Area Under the Curve (AUC):** AUC measures the ability of the model to distinguish between positive and negative classes, summarizing the model’s performance across all classification thresholds. A higher AUC value indicates better discrimination capability.
- **Accuracy:** Accuracy evaluates the proportion of correctly classified instances among the total samples, offering an overall measure of how well the model performs on the dataset, which is calculated as

$$\mathrm{Accuracy}=\frac{TP+TN}{TP+TN+FP+FN}$$

- **Recall:** Recall, also known as sensitivity or true positive rate, quantifies the proportion of actual positive instances correctly identified by the model, focusing on minimizing false negatives. It is defined as

$$\mathrm{Recall}=\frac{TP}{TP+FN}$$

- **Precision:** Precision measures the proportion of correctly predicted positive instances among all instances predicted as positive, reflecting the model’s ability to minimize false positives, which is obtained by

$$\mathrm{Precision}=\frac{TP}{TP+FP}$$

- **F1-Score:** The F1 score is the harmonic mean of precision and recall, balancing the trade-off between these two metrics. It is particularly useful when the dataset is imbalanced.

$$F1=2\cdot \frac{\mathrm{Precision}\cdot \mathrm{Recall}}{\mathrm{Precision}+\mathrm{Recall}}$$

- **Mean Squared Error (MSE):** MSE is a regression metric that calculates the average squared difference between predicted and true values. It measures the model’s prediction accuracy for continuous outputs, where smaller values indicate better performance. The formula of MSE is”

$$\mathrm{MSE}=\frac{1}{n}\overset{n}{\underset{i=1}{\sum }}{\left(y_{i}-{ŷ}_{i}\right)}^{2}$$

where *y*_*i*_ is the true value, ${ŷ}_{i}$ is the predicted value, and *n* is the number of samples.

AUC, accuracy, recall, precision, and F1-score are utilized together to evaluate the models’ performance on fine-tuning tasks comprehensively. At the same time, MSE evaluates the effectiveness and performance of encoder pretraining.

### Details of Core Encoder Model and CoLiM Model

We utilized QCD^50^ to pretrain the core encoder. Leveraging the structural and elemental similarity between gas-phase clusters and metal cores in CMNs, we aimed to train a core encoder model to predict the energy values of gas-phase clusters in a supervised manner. Utilizing a typical GNN (Graph Neural Network) architecture, this model incorporates a linear embedding layer and several CGCNN layers^33^ as the core encoder to learn the high-dimensional features. To enhance the feature extraction process, we employed the skip connection between the input and output of each CGCNN layer, avoiding the traditional linear updating of features:

$$h_{i}^{0}=\mathrm{Embedding}\;\left(G\right),h_{i}^{\left(l+1\right)}=\mathrm{CGCNN}\;\left(h_{i}^{\left(l\right)}\right)+h_{i}^{\left(l\right)}$$

where $h_{i}^{\left(l\right)}$ represents the feature vector of node *i* at layer *l*, **Embedding** and **CGCNN** are the embedding layer and CGCNN layer. The skip connection directly adds the layer’s input to the output, enhancing gradient flow and improving feature extraction. The graph-level representation is obtained through a graph pooling operation:

$$h=\mathrm{Pool}\;\left(h_{0}^{\left(0\right)},h_{1}^{\left(0\right)},...,h_{N}^{\left(0\right)},...,h_{N}^{\left(L\right)}\right)$$

where *N* is the total number of atoms in the graph and **Pool** is the graph pooling operation. Finally, an MLP serves as the regression head to predict the energy value, utilizing softplus as the activation function to ensure nonlinearity in the output layer:

$$E=W_{2}\cdot \mathrm{Softplus}\;\left(W_{1}\cdot h+b_{1}\right)+b_{2}$$

CoLiM utilizes a dual-encoder model structure designed explicitly for the inorganic core-ligand compatibility prediction task. The core encoder adopts the same architecture as the core encoder model, while pretrained UniMol is employed as the ligand encoder:

$$h_{\mathrm{core}}=\mathrm{CoreEncoder}\;\left(G\right),,h_{\mathrm{ligand}}=\mathrm{UniMol}\;\left(L\right)$$

where G is the core coordinate input, L is the ligands SMILES input. Features generated by each encoder are concatenated to form a paired representation, which is then processed through a batch normalization and a dropout layer to enhance model generalization and prevent overfitting:

$$h_{\mathrm{pair}}=\mathrm{Concat}\left(h_{\mathrm{core}},h_{\mathrm{ligand}}\right)$$

$$h_{\mathrm{pair}}=\mathrm{Dropout}\left(\mathrm{BatchNorm}\left(h_{\mathrm{pair}}\right)\right)$$

where Concat is concatenation operation, Dropout and BatchNorm is the dropout layer and batch normalization layer. The final prediction is calculated using a task-specific classification head (MLP):

$$Y=W_{2}\cdot \mathrm{Softplus}\left(W_{1}\cdot h_{\mathrm{pair}}+b_{1}\right)+b_{2}$$

where Softplus is the softplus activation function, calculated as

$$\mathrm{Softplus}\left(x\right)=\ln\left(1+e^{x}\right)$$

### Materials

Acetic acid, formic acid, and high-performance liquid chromatography (HPLC) grade solvents (chloroform and hexane) were purchased from Sigma–Aldrich. All chemicals were used directly without further purification. Cu36 was synthesized according to our previously reported method.^63^

### Synthesis of Cu_20_ and Cu_19_

Cu_20_ and Cu_19_ were obtained via the acid-induced transformation of Cu_36_. Briefly, 10 mg of Cu_36_ was dissolved in 2 mL of chloroform. 5 μL of acetic acid or formic acid was added to synthesize Cu_20_ or Cu_19_, respectively. Then, hexane was added as the antisolvent. Crystals of Cu_20_ and Cu_19_ could be observed within 1 week.

### Characterizations

Single-crystal X-ray diffraction data of Cu_20_ and Cu_19_ were collected on a Bruker D8 Venture diffractometer with a SMART APEX2 area detector (Cu Kα, λ = 1.54184 Å) at 120 K. UV–vis absorption spectra were recorded on a Cary 5000 UV–vis spectrometer. ESI-MS spectra were recorded on a Bruker MicroTOF-II mass spectrometer using chloroform as the solvent.

## Supplementary Material



## Data Availability

The source code and scripts for the core–ligand matching model (CoLiM) are available at https://github.com/JIAYI0012/CoLiM_code.git.

## Notes

Details and hyperparameters about the CoLiM model and baseline models; statistical analysis about the dataset; sample utilized in the external test; CoLiM model’s prediction behavior; crystal data for Cu_19_ and Cu_20_; details of the permutation invariance of ligands encoder; calibration analysis, confidence interval, and confusion matrix of the CoLiM model (PDF)

## Notes

Accession Codes CCDC 2430843–2430844 contain the supplementary crystallographic data for this paper.
