# Supplementary material for: Deep Learning Framework for Atomic-Level Design and Presynthesis Prediction of Coinage-Metal Nanoclusters
Source: ACS Cent Sci. 2026 Jan 8;12(1):88–99. doi: 10.1021/acscentsci.5c01610 (PMC12856671; doi:10.1021/acscentsci.5c01610)
Supplement: Supplementary file 1 [file oc5c01610_si_001.pdf]

Supplementary Information

Deep Learning Framework for Atomic-Level Design and Pre-Synthesis Prediction of Coinage-Metal Nanoclusters

Jiayi Wang,† Chunwei Dong,† Xiaochuan Gou,‡ Shaopeng Fu,‡ Peng Yuan,† Xin Song,† Mohammad Bodiuzzaman,† Mutalifu Abulikemu,† Wanyu Lin,¶ Ren-wu Huang,§ Omar F.Mohammed,† Di Wang,*,‡ and Osman Bakr*,†

†*Center of Excellence for Renewable Energy and Storage Technologies, Division of Physical Science and Engineering, King Abdullah University of Science and Technology (KAUST), Thuwal 23955-6900, Kingdom of Saudi Arabia*

‡*Center of Excellence for Generative AI, Division of Computer, Electrical and Mathematical Sciences and Engineering (CEMSE), King Abdullah University of Science and Technology (KAUST), Thuwal 23955-6900, Kingdom of Saudi Arabia*

¶*Department of Data Science and Artificial Intelligence, Department of Computing, The Hong Kong Polytechnic University, Hong Kong, China*

§*Henan Key Laboratory of Crystalline Molecular Functional Materials, College of Chemistry, Pingyuan Laboratory, Zhengzhou University, Zhengzhou 450001, P.R. China*

E-mail: di.wang@kaust.edu.sa; osman.bakr@kaust.edu.sa

Details of CGCNN and UniMol

In this work, we utilize CGCNN[1] and UniMol[2] as the core and ligands encoder in CoLiM.

CGCNN

CGCNN employs a graph-based representation where atoms and their connections are directly encoded as nodes and edges, capturing the fundamental geometrical and chemical relationships inherent in the crystal structure. CGCNN transforms these graphs using graph convolutional layers. The convolution layers **Conv** utilize a novel graph convolution function that updates node features by considering both their features and those of their neighbors, weighted by the bond characteristics:

$$v_i^{(t+1)} = \mathrm{Conv}(v_i^{(t)}, v_j^{(t)}, u_{(i,j)k}) \quad \text{for all } (i,j)k \in G.$$

where v_i is the feature vector of node i in $i-th$ iteration and $u_{(i,j)k}$ represents the feature edge vector of $k-th$ edge between node v_i and v_j.

Following **T** convolutional iterations, the network autonomously acquires the feature vector $v_{\mathrm{i}}{}^{(\mathrm{T})}$ for each atom, progressively integrating information from its adjacent environment. Subsequently, a pooling layer is employed to generate a comprehensive feature vector for the entire crystal. This vector is derived through a pooling function, effectively summarizing the distributed features into a unified representation.

$$v_c = \mathrm{Pool}\left(v_0^{(0)}, v_1^{(0)}, \ldots, v_N^{(0)}, \ldots, v_N^{(T)}\right)$$

where N is the total number of atoms in the graph and Pool is the graph pooling function.

In addition to the convolutional and pooling layers, two fully-connected hidden layers with the depth of L1 and L2 are added to capture the complex mapping between crystal structure and property. Finally, an MLP served as the output layer is used to connect the

L2 hidden layer to predict the target property $\hat{y}$.

UniMol

UniMol is a molecule representation learning (MRL) framework that utilizes transformers as the backbone model, as it could learn the possible long-range interaction from the fully connected atomic graphs. The primary goal of UniMol is to address the limitations of current MRL methods by incorporating 3D information directly into the learning process. This approach aims to significantly improve the performance of molecular property prediction and enable precise 3D geometry generation, which is crucial for tasks such as drug design and protein-ligand binding. UniMol takes two inputs, atom types, and atom coordinates. The atom representation is then initialized by embedding layers from the provided atom type, while atomic pair representation is obtained through positional encoding. To update pair representation, they use atom-to-pair communication via the result of the multihead Query-Key product in self-attention. Formally, the update of $\rangle|$ pair representation is denoted as:

$$q_{ij}^{l+1} = q_{ij}^{l} + \left\{ \frac{Q_i^{l,h}(K_j^{l,h})^T}{\sqrt{d}} \right\}_{h\in[1,H]}$$

where q_{ij}^l is the pair representation of atom pair ij in l-th layer, H is the number of attention heads, d is the dimension of hidden representations, and $Q_i^{l,h}(K_j^{l,h})$ is the Query (Key) of the i-th (j-th) atom in the l-th layer h-th head. These two representations communicate with each other in the self-attention module:[2]

$$\text{Attention}(Q_i^{l,h}, K_j^{l,h}, V_j^{l,h}) = \text{softmax}\left(\frac{Q_i^{l,h}(K_j^{l,h})^T}{\sqrt{d}} + q_{ij}^{l-1,h} \right) V_j^{l,h},$$

where $V_j^{l,h}$ is the Value of the i-th atom in the l-th layer h-th head. By utilizing 209 million molecule conformations, the model is trained on 3D position recovery and masked atom prediction tasks, enhancing its ability to learn 3D spatial representations. In CoLiM, We

utilized a pre-trained UniMol block to generate ligands representation by taking SMILES of ligands as input.

Details of Model Training

This section describes the encoder pre-training and CoLiM finetuning setups in detail.

Encoder Pretraining

The core encoder model was trained on the energy prediction tasks from QCD to learn the representation of the gas-phase nanocluster. The model comprises an embedding layer, a GNN block, and a regression head. We first benchmarked different GNN models as the core encoder block on QCD, as shown in Table S1. Considering the size of QCD dataset, we select CGCNN,[1] Graph Attention Networks (GAT),[3] , Graph Isomorphism Network (GIN),[4] Graphormer,[5] SchNet,[6] DimNet++[7] and Gemnet[8] as baseline models. We chose two tasks from QCD to evaluate model performance: formation energy prediction and HOMO-LUMO gap prediction. We use mean-squared-error (MSE) as the evaluation metric, which is calculated as:

$$\mathrm{MSE} = \frac{1}{n}\sum_{i=1}^{n}(y_i - \hat{y}_i)^2$$

where n is the number of data in one batch, y_i represents the ground truth value and $\hat{y}_i$ is the predicted value. CGCNN archived the lowest MSE, which is thereby selected as our core-encoder architecture. The hyperparameters used for training CGCNN are shown in Table S2.

Table S1: Benckmark on QCD

Model	E_{form}(MSE)	$E_{\text{HOMO-LUMO}}$(MSE)
CGCNN	0.052	0.117
GAT	0.377	0.180
GIN	0.177	0.133
Graphormer	0.103	0.144
SchNet	0.130	0.130
DimNet++	0.078	0.129
GemNet	0.060	0.124

Table S2: Hyperparameters of Core Encoder Model

Hyperparameter	
Epochs	150
Batch-size	256
Learning Rate	0.01
Scheduler	MultiStepLR
LR milestones	50
Optimizer	SGD
Momentum	0.9
Weight-decay	1e-5
Atom-fea-len	64
Hidden-fea-len	128
N-convs	3
Activation Function	Softplus

Finetuning in CoLiM

CoLiM is optimized on our self-constructed core-ligands matching dataset in the finetuning stage. It takes two inputs: a coordinate file of core configuration and a SMILE representation of ligands. Core and ligand representation is maintained through the pre-trained coder encoder and pre-trained UniMol, respectively, which further concatenates to generate pair representation. Final predictions are calculated by the task-specific classification head (MLP). Cross-entropy is utilized as the loss function, which measures the discrepancy between the predicted probabilities and the actual class labels, providing a quantitative assessment of the model's prediction accuracy:

$$Loss = -\left(y\log(\hat{y}) + (1-y)\log(1-\hat{y})\right)$$

We employed the area-under-the-curve (AUC) and accuracy as the primary evaluation metrics to assess model performance. In addition to these, we also compared the model's performance, both with and without pre-trained encoder, using other metrics such as the F1 score, precision, and recall. The model is optimized using SGD with hyparameter shown in Table S3. All parameter in CoLiM model are being optimized during finetuning except for the UniMol encoder. The model with best validation AUC is selected for evaluation in the set set.

Table S3: Hyperparameters of CoLiM finetuning

hyperparameter	CoLiM
Epochs	100
Batch-size	64
Learning Rate	0.01
Scheduler	MultiStepLR
LR milestones	60
Optimizer	SGD
Momentum	0.9
Wd	1e-3

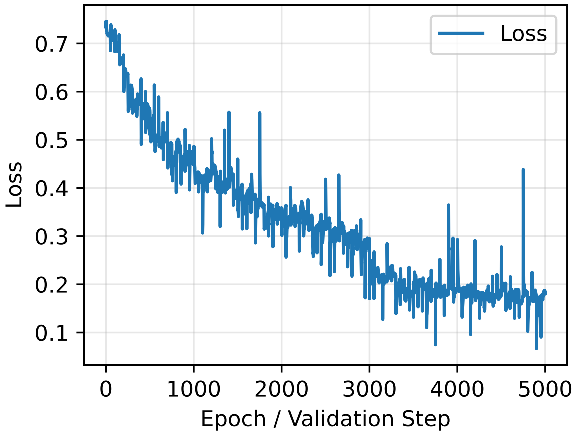

(a) Train Loss Convergence

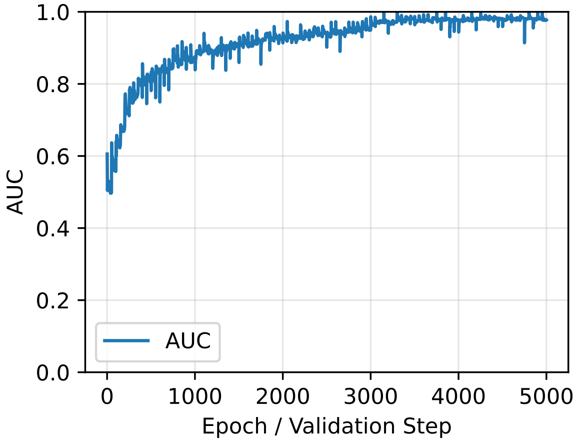

(b) Train AUC Convergence

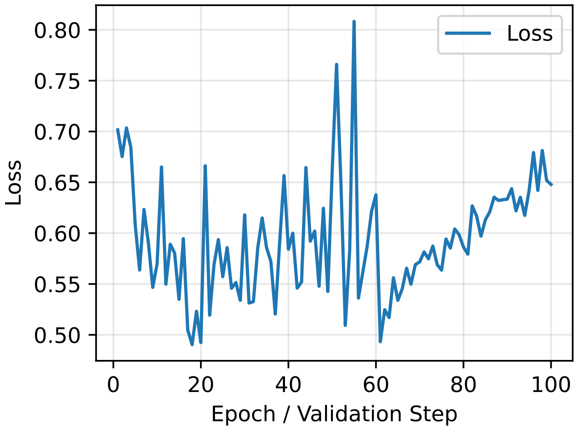

(c) Val Loss Convergence

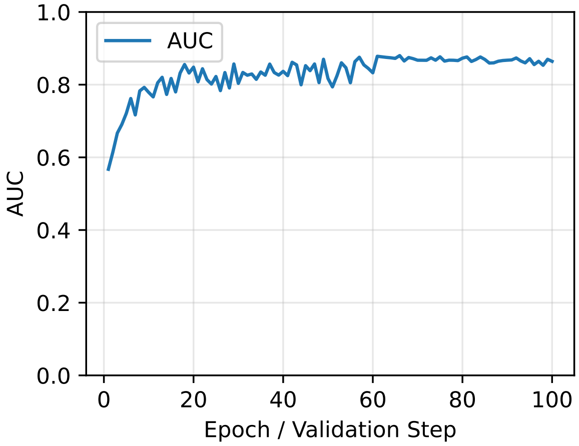

(d) Val AUC Convergence

Figure S1: Convergence Curve

Baseline Methods Settings

In this section, we detail the experimental setups used to compare benchmark models with the CoLiM framework, specifically including models such as SOAP (Smooth Overlap of Atomic Positions), MBTR (Many-Body Tensor Representation), and CGCNN. These comparisons are designed to evaluate the performance and effectiveness of CoLiM against established methodologies in our tasks.

SOAP

The SOAP[9] is a method used in computational chemistry and materials science to quantitatively analyze and compare local atomic environments across different molecular or crystalline structures. It involves constructing a smooth, continuous density function for each atomic environment using Gaussian distributions centered on the atoms. These density functions are then compared by calculating the overlap integrals, which are made rotationally invariant through a transformation into a power spectrum derived from spherical harmonics. This approach allows SOAP to capture subtle differences in local geometries and provides a robust, high-resolution descriptor invariant to identical atoms' rotations and permutations. These characteristics make SOAP particularly useful for applications in material discovery, machine learning model input features, and molecular dynamics analysis, where precise and transferable descriptions of atomic structures are essential. To evaluate the performance of SOAP in our core-ligand matching task, we initially generated SOAP vectors for both cores and ligands using the DScribe package.[10] Given that the length of a SOAP vector varies with the number of elements in the system,[9] we implemented sequence padding to standardize the length of the SOAP vectors, ensuring uniformity across all samples. The hyperparameters used to generate the SOAP vector are summarized in Table S4. XGBoost[11] makes predictions through concatenated vectors with uniform length.

Table S4: Parameters of SOAP generator

rcut	nmax	lmax	σ	average
6.0	8	6	0.2	inner

MBTR

The Many-Body Tensor Representation (MBTR)[12] is an advanced descriptor used in computational chemistry and materials science to comprehensively characterize the atomic and molecular systems' structure. This method constructs high-dimensional descriptors by considering the relationships between atoms in multiple body orders, effectively capturing the geometry and chemical environment at different scales. MBTR first calculates distributions of specific properties, such as distances (two-body terms), angles (three-body terms), and dihedral angles (four-body terms) among atoms. These distributions are then transformed into a tensor representation through a systematic approach that includes weighting and discretization to facilitate the capture of critical structural details.[12] The versatility and depth of information provided by MBTR make it highly effective for tasks that require precise material characterization, such as predicting material properties, facilitating machine learning models in materials informatics, and identifying novel materials with desired features. The experimental settings for MBTR are conceptually similar to those used for SOAP, as the parameters are shown in Table S5.

Table S5: Parameters of MBTR

function	$grid_{min}$	$grid_{max}$	n-grid	σ
inverse-distance	0	1	100	0.1
$weighting_{function}$	$weighting_{scale}$	$weighting_{threshold}$	normalization	periodic
exp	0.5	1e-3	l2	False

ACSF

The Atom-Centered Symmetry Functions (ACSF)[13] is a widely used descriptor in computational chemistry and materials science, designed to encode the local atomic environment in a computationally efficient and interpretable manner. ACSF represents the local structure around an atom by constructing symmetry functions that capture radial and angular distributions of neighboring atoms within a cutoff distance. Radial symmetry functions characterize pairwise interactions between atoms based on their distances. In contrast, angular symmetry functions describe three-body interactions by considering the angles formed by an atom and its neighbors. These functions are parameterized to ensure invariance to translations, rotations, and permutations of identical atoms, making ACSF particularly suitable for machine learning tasks. By converting the complex local environment into a compact feature vector, ACSF facilitates tasks such as property prediction, molecular dynamics simulations, and structure optimization.[13] The implementation of ACSF involves tuning hyperparameters such as the cutoff radius, the number of symmetry functions, and their functional forms, which are comparable to those used in MBTR, as shown in TableS6.

Table S6: Parameters of ACSF

rcut	10.0
g2_params	[[1,1], [1,2], [1,3]]
g4_params	[[1,1,1], [1,2,1],[1,1,-1], [1,2,-1]]

GNN models

Since the core-ligands matching task requires two inputs, a metal core graph, and the ligands graph, we compare the GNN methods by utilizing GNN blocks as feature creators. Specifically, we utilize different GNN blocks to generate features of given core and ligand input, similar to the CoLiM process. The obtained representation vectors are then concatenated to generate the pair representations. The final prediction is calculated by a classification head (MLP). We compared CGCNN,[1] Schnet[6] and GIN[4] with CoLiM models. The training setup is shown in Table S7, Table S8, and Table S9.

Table S7: Parameters of CGCNN

Hyperparameter	CGCNN
Epochs	150
Batch-size	256
Learning Rate	0.01
Scheduler	MultiStepLR
LR milestones	50
Optimizer	SGD
Momentum	0.9
Weight-decay	1e-5
Atom-fea-len	64
Hidden-fea-len	128
N-convs	3
Activation Function	Softplus

Table S8: Parameters of Schnet

Hyperparameter	SchNet
Epochs	150
Batch-size	128
Learning Rate	0.0001
Scheduler	MultiStepLR
LR milestones	60
Optimizer	Adam
Weight-decay	1e-5
Hidden-fea-len	128
Num-filters	128
Num-gaussians	50
Num-radial	6
Dropout	0.5

Table S9: Parameters of GIN

Hyperparameter	GIn
Epochs	100
Batch-size	128
Learning Rate	0.0001
Scheduler	MultiStepLR
LR milestones	30
Optimizer	Adam
Weight-decay	1e-3
Hidden-fea-len	300
Out-emb-channel	256
Num-layers	3
Num-head	8
Dropout	0.5

Core-Ligands Matching Dataset

Table S10: Dataset Clusters Size Statistics

Number of Atoms Range	Sample Count
0–100	95
101–200	376
201–300	601
301–400	414
401–500	237
501+	266

Table S11: Core Library Statistics

Number of Element	Sample Count
1	694
2	965
3	303
4+	27

Table S12: Ligands Library Statistics

Number of Ligands Type	Sample Count
1	816
2	775
3	258
4+	140

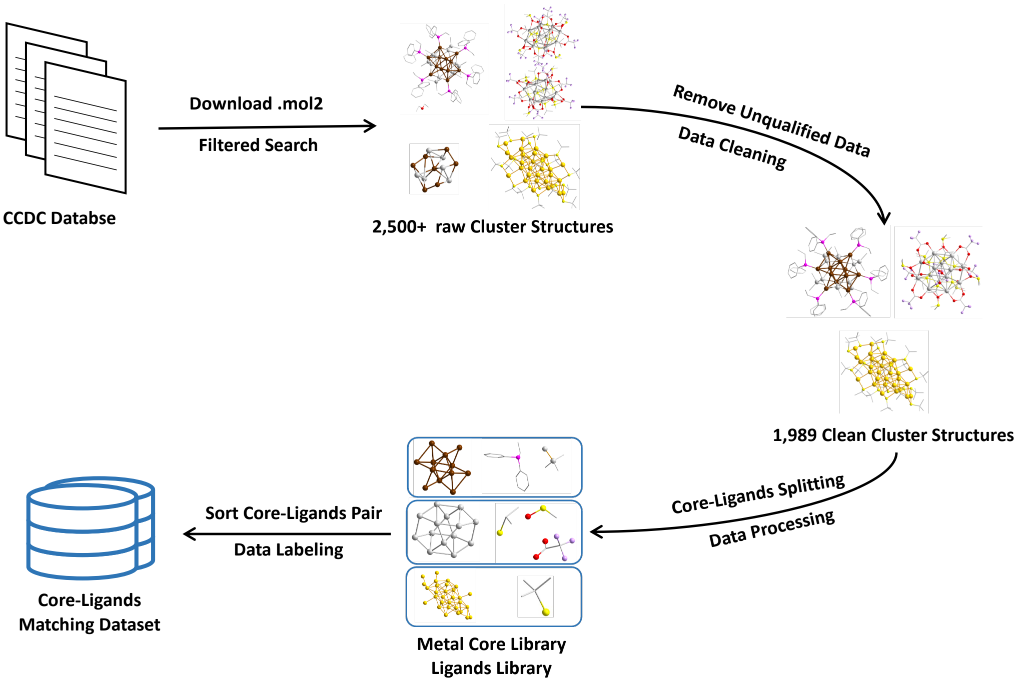

Figure S2: An illustrative diagram showing the process of generating core-ligands matching dataset. 1,989 cluster structures were extracted, cleaned, and split to build the cluster core library and ligands library, which were utilized to create the core-ligands matching dataset through Data Labeling.

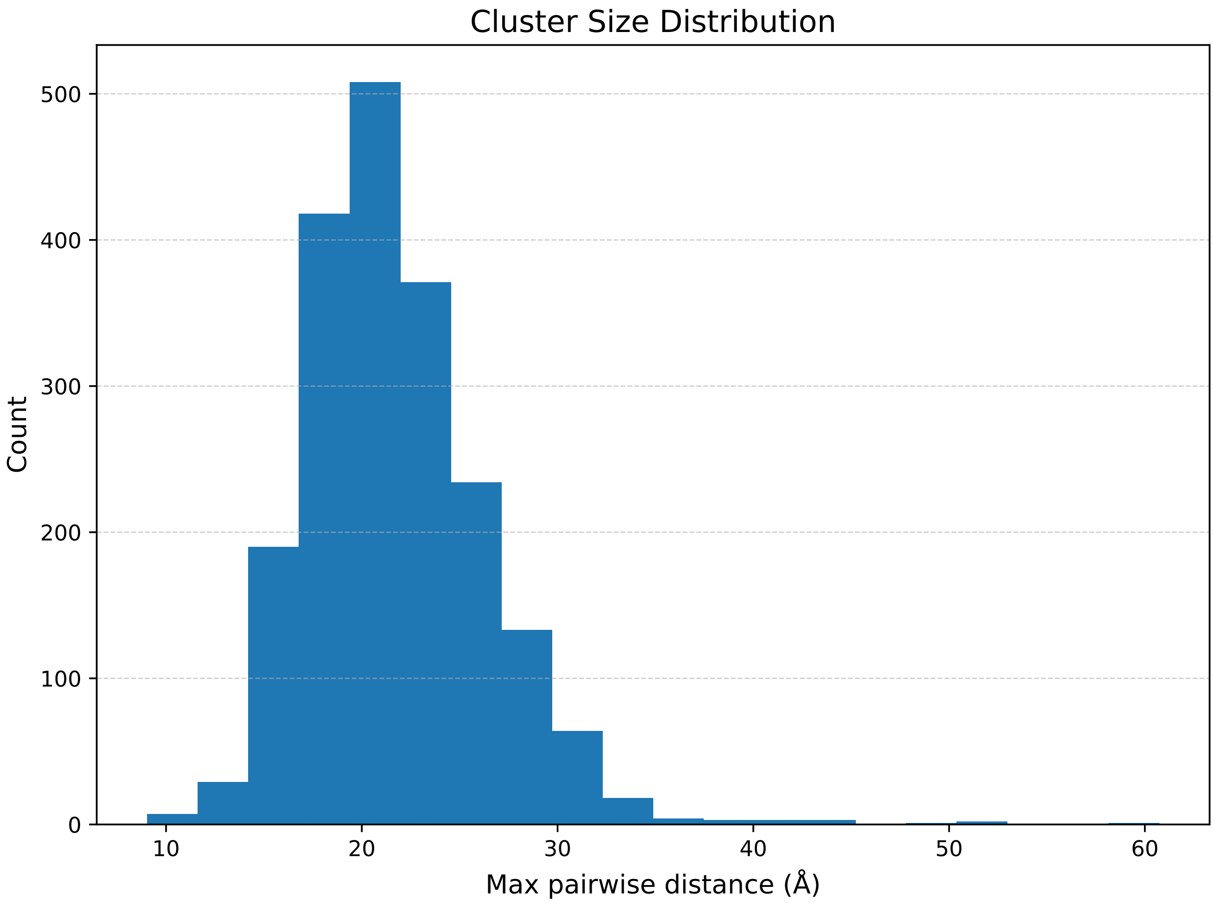

Figure S3: The size distribution of the collected structures, where the diameter is calculated as the maximum distance within the structure.

Table S13: The nuclearity distribution of collected 1,989 nanocluster structures.

Nuclearity Range	Sample Count
4-8	381
9-13	398
14-18	298
19-23	256
24-28	235
29-33	102
34-38	81
39+	238

Table S14: Distribution of nuclearity type

Nuclearity Type	Sample Count
Homonuclearity	694
Heternuclearity	1295

Table S15: Distribution of ligand type

Ligand Type	Sample Count
Phosphines	1084
Thiolate	794
Alkynyl	651
Carboxylates	287
Halides	214
N-hetericycles	182

Test Results Visualization

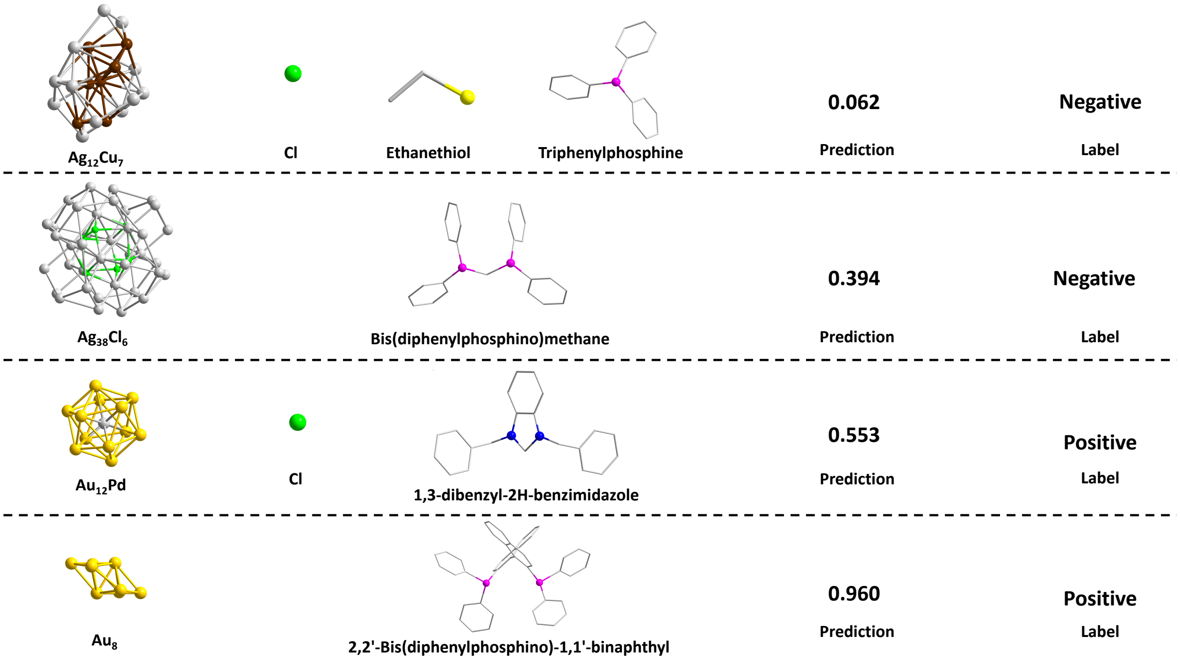

Figure S4: Visualization of four examples in the test set. Logistic for being labeled "can be synthesized" is calculated for a given core configuration and ligands combinations; exceeding 0.5 is regarded as positive pairs, meaning that the model predicts this ligands combination could more likely form a compound consisting of the given core-configuration

External Test

This section describes how we test our model from external data. Since the recently reported clusters are not included in our dataset, we select 10 cluster structures to perform external tests. The compound's full name, the short name, usually the core elements composition, and source papers are shown in Table 7. We use the model for predicting Cu_{19} for external tests.

Table S16: Structures for external test

Cluster	Short	ref
$([(MoO_4{}^{2\text{-}})Ag_{20}(CC^{t}Bu))_8(Ph_2PO_2)_7(tfa)_2]\cdot(tfa–)$	$Ag_{20}Mo$	ref.[14]
$([(MoO_4{}^{2\text{-}})@Ag_{18}(CC^{t}Bu))_8(Ph_2PO_2)_7]\cdot(OH)$	$Ag_{18}Mo$	ref.[14]
$Ag_{20}Cu_{12}$(3,5-bis(trifluoromethyl)phenylacetylene)$_{24}$	$Ag_{20}Cu_{12}$-1	ref.[15]
$Ag_{20}Cu_{12}$(3,5-bis(trifluoromethyl)phenylacetylene)$_{24}$	$Ag_{20}Cu_{12}$-2	ref.[15]
$(Au_{13}(bisNHC)_5Cl_2)Cl_3$	Au_{13}	ref.[16]
$Cu_{26}(Se)_{12}(DPPE)_6$	$Cu_{26}Se_{12}$	ref.[17]
$(Cu_{45}(TBBT)_{29}(TPP)_4(C_4H_{11}N)_2H_{14})^{2+}$	Cu_{45}	ref.[18]
$Cu_{13}(Nap)_3(PPh_3)_7H_{10}$	Cu_{13}	ref.[19]
$(Et_3NH)(Cl@Cu_8(TC_4A)_2(Ph_2SiO_2)_4)(C_3H_6O)_3$	Cu_8	ref.[20]
$(Cu_{34}S_7(RS)_{18}(PPh_3)_4)^{2+}$	Cu_{34}	ref.[21]

Davies-Bouldin index and Calinski-Harabasz index

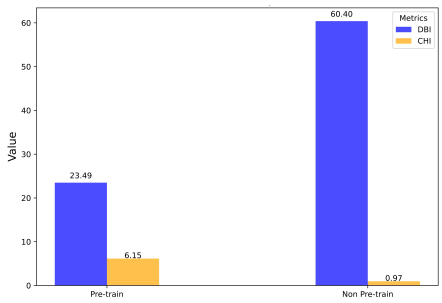

Figure S5: DBI and CHI of CoLiM with/without pre-trained encoder

Case Study on the model's prediction behavior

To gain further insight into which interpretable descriptors drive CoLiM's predictions, we trained a logistic regression surrogate to discriminate high-score from low-score core–ligand pairs on the held-out test set. High-score samples were defined as those with CoLiM predicted probabilities ≥ 0.7 and low-score samples as those with probabilities ≤ 0.3, discarding intermediate cases. Using standardized descriptors (nuclearity, mean_metal, core_size, number_of_ligands, and the fractions of each ligand-family code), the surrogate achieves an accuracy of 0.58 on this subset, with an F1-score of 0.67 and a recall of 0.82 for the high-score class (Table S17).

The learned coefficients (Table S18) reveal clear chemical trends. Among the ligand descriptors, the fraction of ligand family type 2 (Phosphines) has a pronounced positive coefficient (+0.13), indicating that CoLiM is more likely to assign high scores to cores surrounded by Phosphines-rich shells. In contrast, the fraction of ligand family type 6 (Halide) shows the strongest negative coefficient (–0.20), suggesting that such shells are systematically associated with low CoLiM scores.

Regarding the global core descriptors, the coefficients for nuclearity (0.01), core_size (–0.02), mean_metal (0.04), and number_of_ligands (–0.07) are smaller in magnitude, indicating only weak linear trends. The negative coefficient of number_of_ligands suggests that excessively large ligand counts tend to reduce the likelihood of being classified as a high-score pair, while nuclearity and core_size play a comparatively minor role. Overall, these results show that CoLiM's high-confidence predictions are primarily organized by the detailed ligand-family composition around the metal core—favoring chemically reasonable ligand environments.

Table S17: Classification report of the logistic regression surrogate on the high and low-score subset.

Class	Precision	Recall	F1-score	Support
0 (low-score)	0.62	0.33	0.43	160
1 (high-score)	0.57	0.82	0.67	175

Table S18: Logistic regression coefficients for the surrogate model on high and low-score data.

Feature	Coefficient
Halide	−0.196
Phosphines	+0.128
Number of ligands	−0.074
Thiolate	+0.058
Mean_metal	+0.036
N-heterocycles	−0.027
Alkynyl	+0.023
Carboxylates	+0.018
Core_size	−0.016
Nuclearity	+0.014

Case Study

Table S19: Crystal data for Cu_{19} and Cu_{20}.

**Property**	**Cu_{19}**	**Cu_{20}**
**Identification code**	Cu_{19}	Cu_{20}
**Empirical formula**	$C_{163}H_{166}Cl_{22}Cu_{19}O_{12}P_3S_{12}$	$C_{180}H_{186}ClCu_{20}O_{12}PS_{15}$
**Formula weight**	4781.74	4359.40
**Temperature/K**	150.01	150.0
**Crystal system**	trigonal	cubic
**Space group**	R3	Pa-3
a/**Å**	20.610	32.939(3)
b/**Å**	20.610	32.939
c/**Å**	38.621	32.939
$\alpha/°$	90	90
$\beta/°$	90	90
$\gamma/°$	120	90
**Volume/Å^3**	14207.6	35738(11)
**Z**	3	8
$\rho_{\mathbf{calc}}$ **(g/cm^3)**	1.677	1.620
μ **(mm^{-1})**	6.987	4.791
**F(000)**	7206.0	17712.0
**Crystal size (mm^3)**	0.3 × 0.3 × 0.2	0.3 × 0.3 × 0.2
**Radiation**	CuKα (λ = 1.54184)	CuKα (λ = 1.54184)
**2Θ range for data collection/°**	10.172 to 133.176	6.574 to 117.764
**Index ranges**	$-24 \leq h \leq 24$	$-34 \leq h \leq 11$
	$-24 \leq k \leq 24$	$-30 \leq k \leq 36$
	$-45 \leq l \leq 45$	$-32 \leq l \leq 29$
**Reflections collected**	62490	82522
**Independent reflections**	11013 [R_{int} = 0.0468, R_σ = 0.0328]	8554 [R_{int} = 0.0878, R_σ = 0.0480]
**Data/restraints/parameters**	11013/125/677	8554/48/690
**Goodness-of-fit on F^2**	1.020	1.021
**Final R indexes [I≥2σ(I)]**	R_1 = 0.0589, wR_2 = 0.1642	R_1 = 0.0731, wR_2 = 0.2125
**Final R indexes [all data]**	R_1 = 0.0606, wR_2 = 0.1667	R_1 = 0.1343, wR_2 = 0.2761
**Largest diff. peak/hole / eÅ^{-3}**	1.19 / -0.90	1.23 / -0.93
**Flack parameter**	0.10(4)	N/A

Table S20: Summary of some atom–atom distances in Cu_{19} (designed) and Cu_{19} (synthesized).

**Atom–atom**	**Cu_{19} (designed)**	**Cu_{19} (synthesized)**
	Range (Å)	Range (Å)
	Average (Å)	Average (Å)
$Cu_{shell\text{-}1}$–Cl	2.569–2.614	2.419–2.480
	2.580	2.465
$Cu_{shell\text{-}1}$–$Cu_{shell\text{-}2}$	2.889–2.971	2.774–3.176
	2.888	2.973
$Cu_{shell\text{-}2}$–$Cu_{shell\text{-}2}$	2.741–2.741	2.723–2.788
	2.743	2.745
$Cu_{shell\text{-}2}$–$Cu_{shell\text{-}3}$	2.635–2.699	2.713–2.736
	2.655	2.722

Table S21: Potential Ligands and Combinations for Synthesizing Cu_{19}

**Ligands ID**	**Name**	**Abbreviation**
S1	2-Phenylethanethiol	PET
S2	tert-Butyl mercaptan	t-BuS
S3	1-Adamantanethiol	1-AdmSH
S4	4-Methylbenzenethiol	4-MBT
S5	Triphenylphosphine	PPh3
S6	Diphenyl-2-pyridylphosphine	DPPP
D1	Triphenylphosphine; 2-Phenylethanethiol	PPh3;PET
D2	Triphenylphosphine;tert-Butyl mercaptan	PPh3;t-BuS
D3	Triphenylphosphine;1-Adamantanethiol	PPh3;1-AdmSH
D4	Triphenylphosphine;4-Methylbenzenethiol	PPh3;4-MBT
D5	Diphenyl-2-pyridylphosphine;2-Phenylethanethiol	DPPP;PET
D6	Diphenyl-2-pyridylphosphine;tert-Butyl mercaptan	DPPP;t-BuS
D7	Diphenyl-2-pyridylphosphine;1-Adamantanethiol	DPPP;1-AdmSH
D8	Diphenyl-2-pyridylphosphine;4-Methylbenzenethiol	DPPP;4-MBT
T1	Triphenylphosphine;	
	2-Phenylethanethiol;Formic Acid	PPh3;PET;FA
T2	Triphenylphosphine;	
	tert-Butyl mercaptan;Formic Acid	PPh3;t-BuS;FA
T3	Triphenylphosphine;	
	1-Adamantanethiol;Formic Acid	PPh3;1-AdmSH;FA
T4	Triphenylphosphine;	
	4-Methylbenzenethiol;Formic Acid	PPh3;4-MBT;FA
T5	Diphenyl-2-pyridylphosphine;	
	2-Phenylethanethiol;Formic Acid	DPPP;PET;FA

Table S21 continued from previous page

Ligands ID	Name	Abbreviation
T6	Diphenyl-2-pyridylphosphine;	
	tert-Butyl mercaptan;Formic Acid	DPPP;t-BuS;FA
T7	Diphenyl-2-pyridylphosphine;	
	1-Adamantanethiol;Formic Acid	DPPP;1-AdmSH;FA
T8	Diphenyl-2-pyridylphosphine;	
	4-Methylbenzenethiol;Formic Acid	DPPP;4-MBT;FA
T9	Triphenylphosphine;	
	2-Phenylethanethiol;Acetic Acid	PPh3;PET;Ac
T10	Triphenylphosphine; Acid	
	tert-Butyl mercaptan;Acetic	PPh3;t-BuS;Ac
T11	Triphenylphosphine;	
	1-Adamantanethiol;Acetic Acid	PPh3;1-AdmSH;Ac
T12	Triphenylphosphine; Acid	
	4-Methylbenzenethiol;Acetic	PPh3;4-MBT;Ac
T13	Diphenyl-2-pyridylphosphine;	
	2-Phenylethanethiol;Acetic Acid	DPPP;PET;Ac
T14	Diphenyl-2-pyridylphosphine;	
	tert-Butyl mercaptan;Acetic Acid	DPPP;t-BuS;Ac
T15	Diphenyl-2-pyridylphosphine;	
	1-Adamantanethiol;Acetic Acid	DPPP;1-AdmSH;Ac
T16	Diphenyl-2-pyridylphosphine;	
	4-Methylbenzenethiol;Acetic Acid	DPPP;4-MBT;Ac

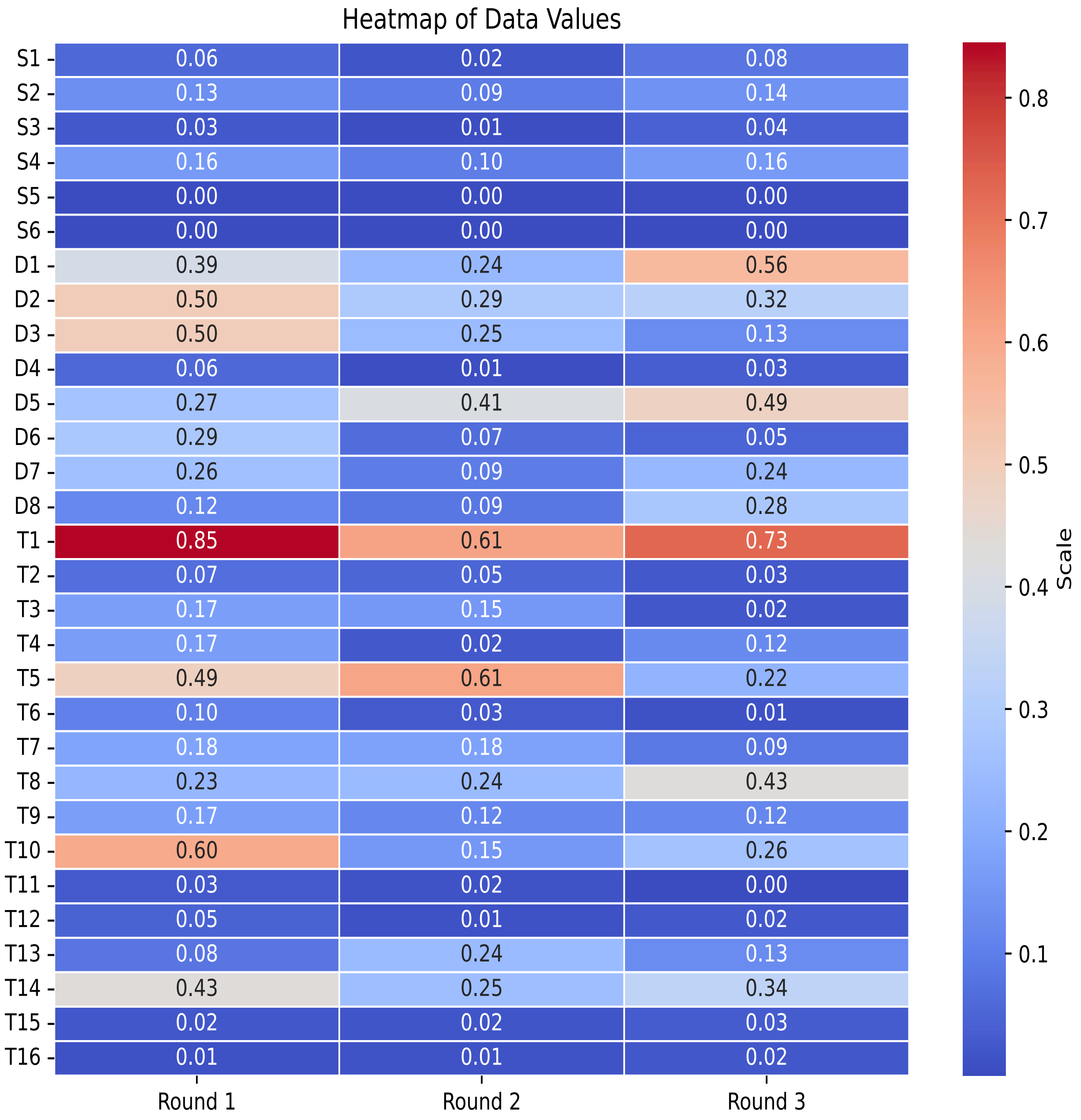

Figure S6: Heatmap of three rounds CoLiM matching prediction utilizing models

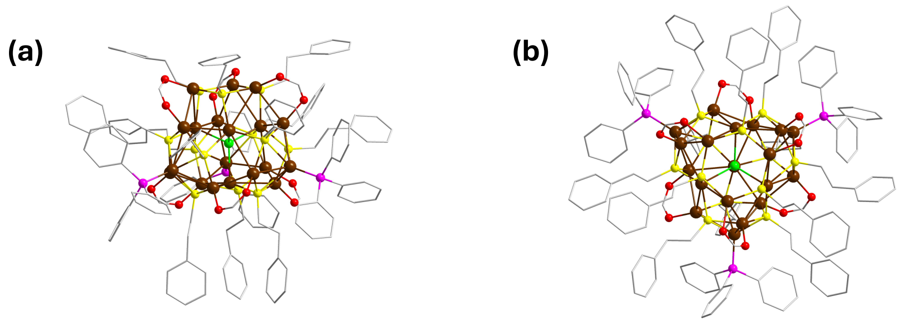

Figure S7: (a) Side and (b) top views of Cu_{19} in a ball-and-stick model. Carbon atoms and bonds are shown in a wire frame model, while all hydrogen atoms in the ligands are omitted for clarity. Brown: Cu; yellow: S; pink: P; red: O; gray: C.

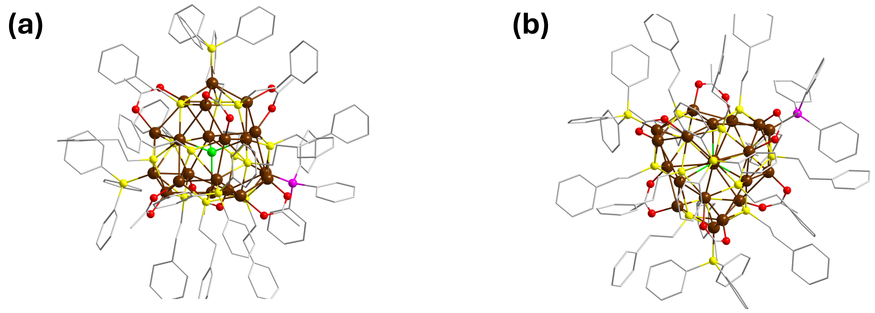

Figure S8: (a) Side and (b) top views of Cu_{20} in a ball-and-stick model. Carbon atoms and bonds are shown in a wire frame model, while all hydrogen atoms in the ligands are omitted for clarity. Brown: Cu; yellow: S; pink: P; red: O; gray: C.

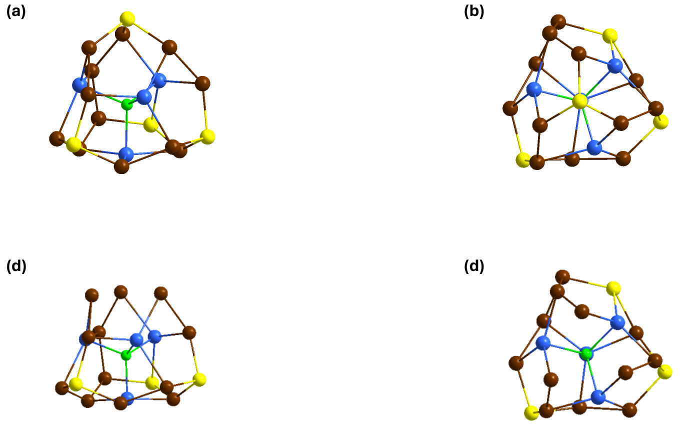

Figure S9: (a,c) Side and (b,d) top views of Cu_{19} and Cu_{20} core structure in a ball-and-stick model. all ligands atoms are omitted for clarity. Green: Cl, Blue: Cu-shell1, Brown: Cu-shell2, yellow: Cu-shell3.

Permutation invariant of Ligand Representation

To evaluate the permutation invariance of UniMol-generated molecular representations for multi-component systems, we constructed multi-SMILES strings containing three distinct fragments and enumerated all possible permutations of their component order ($3! = 6$ permutations per sample). For each permutation, UniMol embeddings were computed and then compared against a fixed base SMILES ordering by calculating the cosine similarity and L2 distance between the corresponding representation vectors. The permutation test was considered successful if the minimum cosine similarity across permutations exceeded a predefined threshold of 0.9999 and the maximum L2 difference remained below 1.0×10^{-4}. Under these criteria, all tested systems passed the permutation-invariance check, with minimum cosine similarities effectively equal to 1.0 and maximum L2 deviations on the order of 10^{-5} or lower, confirming that UniMol representations are numerically stable with respect to the ordering of multi-component SMILES fragments (Table S22).

Table S22: Permutation invariance test of UniMol representations for multi-component SMILES inputs. Sample 1: `[Cl].[S]CCc1ccccc1.P(c1ccccc1)(c1ccccc1)c1ccccc1`; Sample 2: `[S]#[C].[S]C1CCCCC1.P(c1ccccc1)(CCCP(c1ccccc1)c1ccccc1)c1ccccc1`; Sample 3: `CNC.[Na+].[Cl-]`; Sample 4: `CCO.CCO.CCO`.

Sample	n_{parts}	n_{perms}	Min cos sim	Max L2 diff	Cos std	L2 std	Pass
1	3	6	0.9999999999999982	5.2330×10^{-5}	4.15×10^{-8}	1.91×10^{-5}	True
2	3	6	0.9999999999999983	1.1925×10^{-5}	1.11×10^{-16}	8.17×10^{-7}	True
3	3	6	0.9999999999999982	1.9112×10^{-5}	4.26×10^{-8}	1.77×10^{-6}	True
4	3	6	0.9999999999999981	0.0	1.11×10^{-16}	0.0	True

Calibration Analysis and Confidence Interval

Reliability diagram

Predicted probabilities are partitioned into 15 equal-width intervals; for each bin we compute the mean predicted probability and the empirical positive rate, and compare these points to the identity line $y = x$.

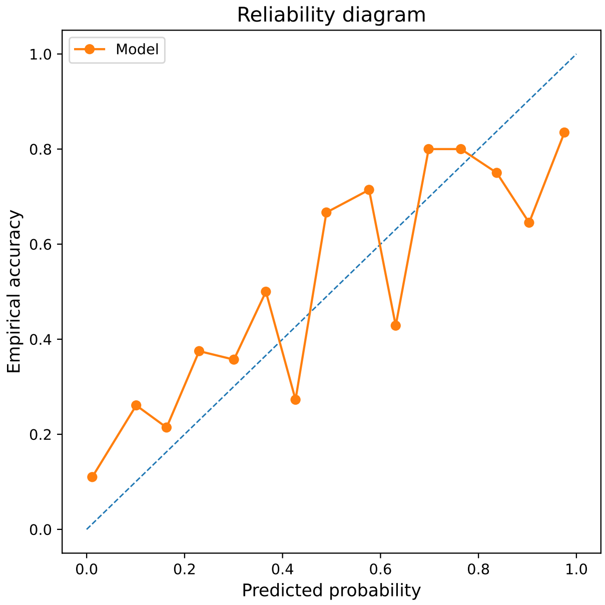

Figure S10: Reliability diagram of CoLiM with bins equal to 15.

Bootstrap Estimation of AUC Confidence Interval and Confusion Matrix

To quantify the uncertainty of the classifier's discrimination performance, we estimated the confidence interval of the ROC AUC using a nonparametric bootstrap procedure. Given the binary ground-truth labels $y \in \{0, 1\}$ and the corresponding continuous prediction scores $\hat{s}$, stratified resampling with replacement was applied to preserve the original class proportions within each bootstrap sample.

In each iteration, positive and negative samples were resampled separately to construct

a new dataset. Bootstrap samples containing only a single class were discarded to ensure that the AUC was well-defined. This procedure was repeated for $B = 3000$ valid bootstrap iterations, resulting in an empirical distribution of the AUC values. The 95% confidence interval was obtained using the percentile method by taking the 2.5% and 97.5% quantiles of the bootstrap distribution.

Using this approach, the resulting 95% bootstrap confidence interval for the ROC AUC is $[0.787, 0.869]$.

Table S23: 95% bootstrap confidence interval of the ROC AUC (3000 stratified bootstrap samples).

Metric	95% CI lower	95% CI upper
ROC AUC	0.787	0.869

Confusion Matrix

Here shows the confusion matrix of CoLiM model with pretrained encoders.

Table S24: Confusion Matrix

	**Pred 0**	**Pred 1**
**Actual 0**	154 (TN)	46 (FP)
**Actual 1**	43 (FN)	154 (TP)

References

(1) Xie, T.; Grossman, J. C. Crystal Graph Convolutional Neural Networks for an Accurate and Interpretable Prediction of Material Properties. *Phys. Rev. Lett.* **2018**, *120*, 145301.

(2) Zhou, G.; Gao, Z.; Ding, Q.; Zheng, H.; Xu, H.; Wei, Z.; Zhang, L.; Ke, G. Uni-Mol: A Universal 3D Molecular Representation Learning Framework. The Eleventh International Conference on Learning Representations. 2023.

(3) Veličković, P.; Cucurull, G.; Casanova, A.; Romero, A.; Liò, P.; Bengio, Y. Graph Attention Networks. 2018; https://arxiv.org/abs/1710.10903.

(4) Xu, K.; Hu, W.; Leskovec, J.; Jegelka, S. How Powerful are Graph Neural Networks? 2019; https://arxiv.org/abs/1810.00826.

(5) Ying, C.; Cai, T.; Luo, S.; Zheng, S.; Ke, G.; He, D.; Shen, Y.; Liu, T.-Y. Do Transformers Really Perform Bad for Graph Representation? 2021; https://arxiv.org/abs/2106.05234.

(6) Schütt, K. T.; Kindermans, P.-J.; Sauceda, H. E.; Chmiela, S.; Tkatchenko, A.; Müller, K.-R. SchNet: A continuous-filter convolutional neural network for modeling quantum interactions. 2017; https://arxiv.org/abs/1706.08566.

(7) Gasteiger, J.; Giri, S.; Margraf, J. T.; Günnemann, S. Fast and Uncertainty-Aware Directional Message Passing for Non-Equilibrium Molecules. 2022; https://arxiv.org/abs/2011.14115.

(8) Gasteiger, J.; Becker, F.; Günnemann, S. GemNet: Universal Directional Graph Neural Networks for Molecules. 2024; https://arxiv.org/abs/2106.08903.

(9) Bartók, A. P.; Kondor, R.; Csányi, G. On representing chemical environments. *Phys. Rev. B* **2013**, *87*, 184115.

(10) Laakso, J.; Himanen, L.; Homm, H.; Morooka, E. V.; Jäger, M. O.; Todorović, M.; Rinke, P. Updates to the DScribe library: New descriptors and derivatives. *The Journal of Chemical Physics* **2023**, *158*.

(11) Chen, T.; Guestrin, C. XGBoost: A Scalable Tree Boosting System. Proceedings of the 22nd ACM SIGKDD International Conference on Knowledge Discovery and Data Mining. New York, NY, USA, 2016; p 785–794.

(12) Huo, H.; Rupp, M. Unified representation of molecules and crystals for machine learning. *Machine Learning: Science and Technology* **2022**, *3*.

(13) Behler, J. Atom-centered symmetry functions for constructing high-dimensional neural network potentials. *The Journal of chemical physics* **2011**, *134*.

(14) Yang, L.; Bigdeli, F.; Yang, X.; Hou, L.-L.; Ma, Y.; Jiang, W.-Y.; Li, X.-H.; Wang, L.-X.; Yang, T.; Wang, K.; Wei, J.; Morsali, A.; Liu, K.-G. Molybdate-Templated Luminescent Silver Alkynyl Nanoclusters: Total Structure Determination and Optical Property Analysis. *Inorganic Chemistry* **2024**, *63*, 7631–7639, PMID: 38625102.

(15) Deng, G.; Malola, S.; Ki, T.; Liu, X.; Yoo, S.; Lee, K.; Bootharaju, M. S.; Häkkinen, H.; Hyeon, T. Structural Isomerism in Bimetallic Ag20Cu12 Nanoclusters. *Journal of the American Chemical Society* **2024**, *146*, 26751–26758, PMID: 39292876.

(16) Yi, H.; Osten, K. M.; Levchenko, T. I.; Veinot, A. J.; Aramaki, Y.; Ooi, T.; Nambo, M.; Crudden, C. M. Synthesis and enantioseparation of chiral Au13 nanoclusters protected by bis-N-heterocyclic carbene ligands. *Chem. Sci.* **2021**, *12*, 10436–10440.

(17) Zhang, C.; Si, W.-D.; Wang, Z.; Dinesh, A.; Gao, Z.-Y.; Tung, C.-H.; Sun, D. Solvent-Mediated Hetero/Homo-Phase Crystallization of Copper Nanoclusters and Superatomic Kernel-Related NIR Phosphorescence. *Journal of the American Chemical Society* **2024**, *146*, 10767–10775, PMID: 38591723.

(18) Alamer, B. et al. Planar Core and Macrocyclic Shell Stabilized Atomically Precise Copper Nanocluster Catalyst for Efficient Hydroboration of C–C Multiple Bond. *Journal of the American Chemical Society* **2024**, *146*, 16295–16305, PMID: 38816788.

(19) Bodiuzzaman, M.; Murugesan, K.; Yuan, P.; Maity, B.; Sagadevan, A.; Malenahalli H, N.; Wang, S.; Maity, P.; Alotaibi, M. F.; Jiang, D.-e.; Abulikemu, M.; Mohammed, O. F.; Cavallo, L.; Rueping, M.; Bakr, O. M. Modulating Decarboxylative Oxidation Photocatalysis by Ligand Engineering of Atomically Precise Copper Nanoclusters. *Journal of the American Chemical Society* **2024**, *146*, 26994–27005, PMID: 39297671.

(20) Qin, H.-N.; He, M.-W.; Wang, J.; Li, H.-Y.; Wang, Z.-Y.; Zang, S.-Q.; Mak, T. C. W. Thiacalix[4]arene Etching of an Anisotropic Cu70H22 Intermediate for Accessing Robust Modularly Assembled Copper Nanoclusters. *Journal of the American Chemical Society* **2024**, *146*, 3545–3552, PMID: 38277257.

(21) Li, S.; Wu, Q.; You, X.; Ren, X.; Du, P.; Li, F.; Zheng, N.; Shen, H. Anchoring Frustrated Lewis Pair Active Sites on Copper Nanoclusters for Regioselective Hydrogenation. *Journal of the American Chemical Society* **2024**, *146*, 27852–27860, PMID: 39352212.
